# Sequential Treatment Application Robot (STAR) for high-replication marine experimentation

**DOI:** 10.1016/j.ohx.2024.e00524

**Published:** 2024-03-28

**Authors:** I.C. Enochs, N. Soderberg, A.M. Palacio-Castro, K. Eaton

**Affiliations:** a Ocean Chemistry and Ecosystems Division, Atlantic Oceanographic and Meteorological Laboratory, NOAA, 4301 Rickenbacker Cswy, Miami, FL 33149, USA; b Cooperative Institute for Marine and Atmospheric Studies, University of Miami, 4600 Rickenbacker Cswy, Miami, FL 33149, USA

**Keywords:** Automation, Marine experimentation, Multiple stressors, Coral reefs, Robotics

## Abstract

Marine organisms are often subject to numerous anthropogenic stressors, resulting in widespread ecosystem degradation. Physiological responses to these stressors, however, are complicated by high biological variability, species-specific sensitivities, nonlinear relationships, and countless permutations of stressor combinations. Nevertheless, quantification of these relationships is paramount for parameterizing predictive tools and ultimately for effective management of marine resources. Multi-level, multi-stressor experimentation is therefore key, yet the high replication required has remained a logistical challenge and a financial barrier. To overcome these issues, we created an automated system for experimentation on marine organisms, the Sequential Treatment Application Robot (STAR). The system consists of a track-mounted robotic arm that sequentially applies precision treatments to independent aquaria via syringe and peristaltic pumps. The accuracy and precision were validated with dye and spectrophotometry, and stability was demonstrated by maintaining corals under treatment conditions for more than a month. The system is open source and scalable in that additional treatments and replicates may be added without incurring multiplicative costs. While STAR was designed for investigating the combined impacts of nutrients, warming, and disease on reef-building corals, it is highly customizable and may be used for experimentation involving a diverse array of treatments and species.

## Graphical abstract

**Figure:**
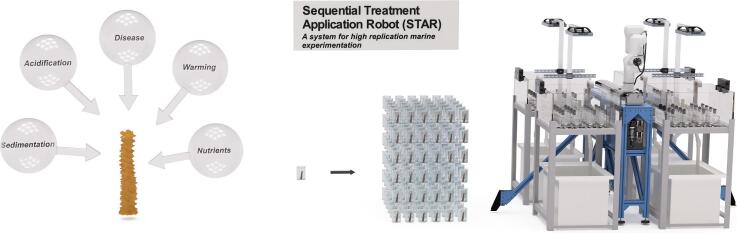

**Specifications table:** 

Hardware name	Sequential Treatment Application Robot (STAR)
Subject area	• Biological sciences (e.g., microbiology and biochemistry) • Environmental, planetary and agricultural sciences • Educational tools and open source alternatives to existing infrastructure
Hardware type	• Biological sample handling and preparation
Closest commercial analog	*“No commercial analog is available.”*
Open source license	Creative Commons Attribution-ShareAlike license
Cost of hardware	$16,939.66
Source file repository	DOI: 10.5281/zenodo.10048123 https://zenodo.org/doi/10.5281/zenodo.10048123

## 1 Hardware in context

Marine ecosystem degradation has become commonplace due to myriad anthropogenic stressors [1]. If we hope to manage and predict the persistence of marine resources, it is imperative that we characterize the stressor-driven responses of marine organisms. While some data may be gleaned from the physiology of species inhabiting marginal environments, from paleoproxies, and from carefully collected time series, these approaches are not without their limitations [2]. Causality is difficult to establish, and treatment levels/combinations can be difficult to identify and replicate. A complementary methodology is the exposure of key species to controlled conditions in a laboratory environment.

Wet lab experimentation, however, is not without its challenges. Precise manipulation of treatment conditions can be difficult and costly, especially when applied at multiple levels. In addition to the application of stressors, one of the biggest difficulties of designing lab-based marine experiments is independent replication [3]. High variability in biological responses is common, driven by factors that include pre-exposure to stress [4], nutrition and fitness [5], genetics [6], as well as differences among the diverse consortia of organisms that comprise the holobiont of a perceived individual [7]. This variability can make signal detection challenging and high replication may be required to characterize subtle responses.

Furthermore, biological sensitivities are often investigated using binary (high vs. none) treatments or even experimental designs with categorical (high vs. mid vs. low) levels of stress. In real life, however, these same stressors present as a continuum (e.g., nutrient concentrations, acidification). Organismal and ecological responses can be non-linear, with tipping points past which individuals may not be able to maintain homeostasis or populations may not be able to persist [8]. These nonlinear responses may obfuscate the results of simplistic experimental studies, thereby necessitating greater numbers of treatment levels. Nevertheless, these thresholds are critically relevant to policies which seek to implement environmentally effective management targets that minimally curtail human activity.

Compounding these issues, stressors seldom occur in isolation and responses to multiple co-occurring stressors are often complex. The combined impacts of these stressors on individual species may be additive, antagonistic, or even synergistic, meaning that studying stressors in isolation is insufficient [8]. While single stressor studies may require high replication, multi-stressor studies exponentially increase sample sizes as additional factors lead to even greater numbers of treatment combinations. All of this can lead to significant cost increases, more complicated infrastructure, and potential logistical issues. The need to overcome the aforementioned issues has prompted various treatment prioritization and statistical solutions (see reduced, fractional, and collapsed designs) [2], [9]. As researchers seek to explain and forecast the real-world manifestation of stressors on marine ecosystems, expanding the scale of complex multifactor designs is unavoidable.

**Fig. 1:**
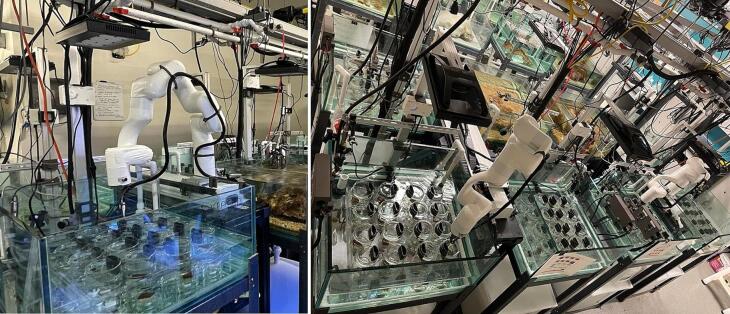
Sequential Treatment Application Robot (STAR), applying unique experimental treatments to an array of beakers containing coral samples.

A solution to overcome the aforementioned challenges lies in robotic automation and the temporal interruption of treatment application. Here we describe a novel approach to solving the need for high-replication, multi-level, multi-stressor marine experimentation, the Sequential Treatment Application Robot (STAR, Fig. 1). The STAR system was developed to test the effects of elevated nutrients on the physiology and susceptibility of corals to Stony Coral Tissue Loss Disease (SCTLD), under multiple temperatures. This system consists of a robotic arm that sequentially applies precision nutrient treatments and one of two water sources (e.g., SCTLD-infected and healthy) to an array of individual beakers, each containing a single independent coral specimen not in contact with any of the other specimens in the experiment.

## 2 Hardware description

• Marine ecosystems and organisms are subject to numerous co-occurring stressors, influencing their form and function.
• Experimentation involving multiple stressor combinations and levels necessitates high replication, which can be logistically difficult and costly.
• The STAR system sequentially administers unique treatments to independent aquarium chambers, facilitating experiments that were previously impossible.
• The STAR consists of a robotic arm and linear track, connected to a precision dosing device, controlled through a graphical user interface.

**Fig. 2:**
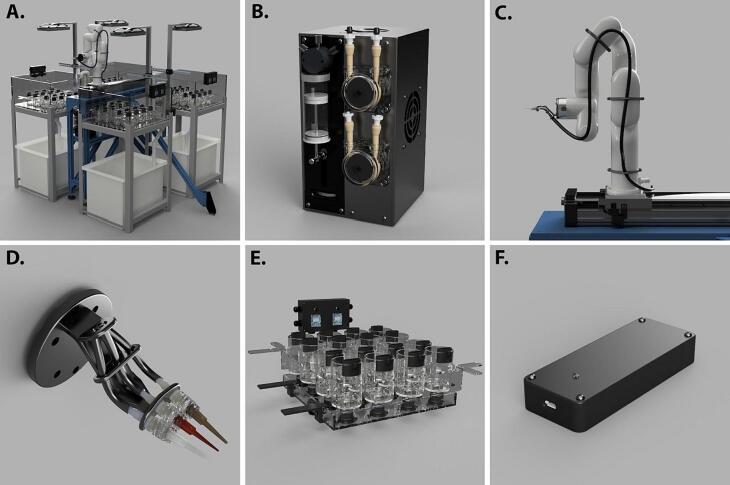
Renderings of the Sequential Treatment Application Robot (STAR) including A. The complete STAR system positioned in a four tank, 64 beaker setup; B. The doser housing with one syringe and two peristaltic pumps; C. The robotic arm, linear track, and tube routing system; D. The end effector with three injection tips; E. The stir system with individual beakers, fluid mixing injection ports, underlying stir plates, and controller box; F. cellular-enabled watchdog.

The STAR system consists of a 6-axis robotic arm affixed to a 700 mm linear track, allowing it to transit back and forth, between four large aquariums (Fig. 2A). A dosing system, comprised of a single high-accuracy syringe pump and two high-flow peristaltic pumps (Fig. 2B), is connected to tubing that is routed along the arm and track (Fig. 2C) to a custom tool at the end of the arm (an end effector, Fig. 2D) which allows the outflow to be directed at a target. The robotic arm moves between glass beakers arranged in each aquarium, sequentially applying treatments. Each beaker sits in a temperature-controlled water bath and contains a stir bar over a stir plate that facilitates mixing and proper aeration (Fig. 2E). Data are logged and uploaded online using a cellular-enabled watchdog device (Fig. 2F), which also alerts users if any errors are encountered. Each of the subsystems within the STAR are customizable for different applications, including tank size and numbers of replicates, as well as stressor levels and combinations, water sources, volumes, and dosing speeds.

### 2.1 System components

Robot system - A 6-axis robot arm (xArm 6, UFACTORY), with 700 mm reach and 6 kg potential payload, is mounted to a linear track that provides an additional 700 mm of travel. A custom end effector is mounted to the distal surface of the arm, directing three nozzles to a focal point beyond the arm’s reach. Tubes extend back from the nozzles, and are routed along the arm using 3D printed guides, ultimately terminating at the dosing system. Communication with the arm is provided through a control box (AC powered), which connects to a computer via an ethernet connection.

**Fig. 3:**
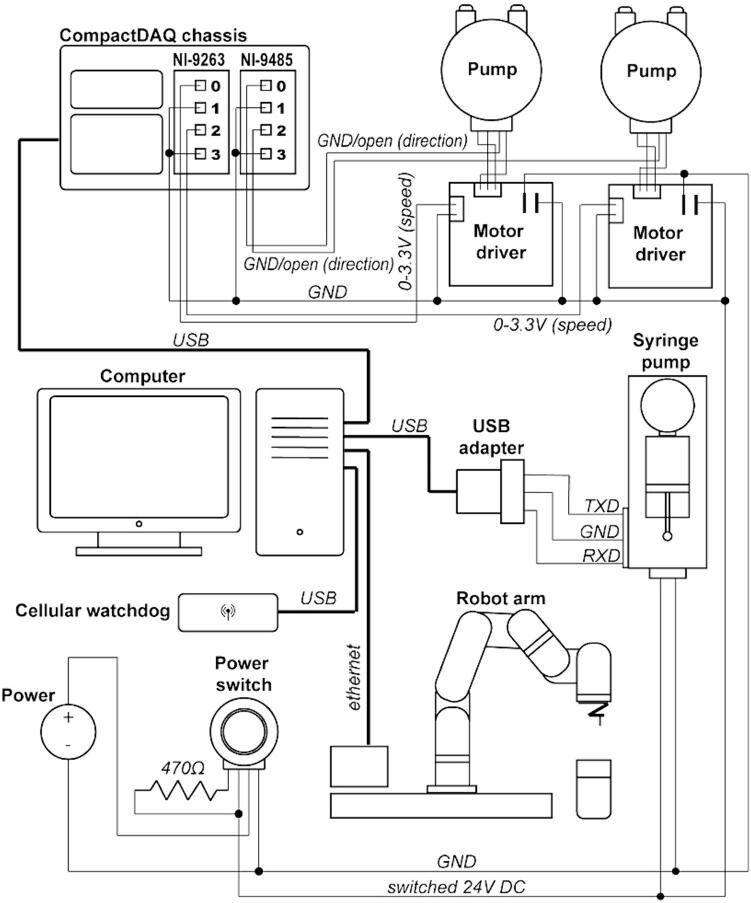
Diagram of electrical connections in the STAR system. The peristaltic pumps are controlled by motor drivers, connected to a computer through a CompactDAQ chassis. The robot arm and syringe pump are connected to the computer via ethernet and a serial/USB adapter, respectively. The cellular watchdog is connected to the computer through an additional USB connection.

Dosing system and control hardware - The dosing system is housed in a custom 3D-printed box and is powered by a 24 V 2.5A DC power supply. The system contains a single syringe pump (Kloehn v6, Norgren) and two brushless peristaltic pumps (A201BX, Anko) plumbed to the arm and three injection tips on the end effector. The syringe pump is controlled via serial communication through a serial adapter card (Kloehn 23438, Norgren), which is connected through a serial/USB adapter to a computer (Fig. 3). The syringe pump may be configured with different volume syringes and rotary valves to control flow speeds, volumes, and directionality. For the purposes of testing, a 5 mL syringe and 6-way distribution valve were used. The peristaltic pumps are each controlled by brushless motor controllers (Anko). Each driver is connected to a voltage output module (0–3.3 V, NI-9263, National Instruments) to control speed, and a relay module (NI-9485, National Instruments) which selectively connects a pin on the motor driver to ground to control pump direction (dispense vs. aspirate fluid, Fig. 3). Each control module is seated into a CompactDAQ chassis (National Instruments) that is connected to a computer via USB. The electronic components in the box are continuously cooled by a 24 V computer fan. Power is switched on and off using a latching rugged pushbutton with an LED connected to switched power through a 470 Ω resistor (Fig. 3).

Stir system - Samples are contained in 4x4 arrays of beakers that are held in position with a laser cut acrylic spacer. As presently designed, each beaker is 600 mL, though different sizes could be used provided that spacers and movement paths are adjusted to accommodate their diameter. Each beaker contains a stir bar, an acrylic sample stand above that stir bar, and a 3D printed injection ports that ensures that the newly injected fluid treatments are fully introduced/mixed into the beaker, rather than pouring off the surface. An array of 16 fan motors, with their blades removed, are affixed to a laser-cut acrylic armature using 3D printed motor attachment base and bolts. A 3D-printed spacer holds a pair of magnets in place on top of each motor, with opposite poles facing upward. These, in turn, spin a stir bar placed in each overlying glass beaker when powered. The 16 stir motors are connected to two custom printed circuit boards (PCB’s) in a 3D printed controller box and the speed is controlled using a Teensy 3.5, and N-channel MOSFETs. Speeds are programmed using an infrared remote control and receiver, and are displayed on an OLED screen with software derived from the SAS and SASe user interfaces [10], [11].

Aquarium system - The aquarium system is employed as a water bath for temperature control of the beakers. Its construction and operation are described by Enochs et al. [12]. Any aquarium or water bath could be fit to serve this function, and the system could theoretically be run without a tank subsystem provided that temperature control and water spillover were not a concern or were managed using other means.

Cellular watchdog - While not required for the operation of the STAR system, error and treatment analytics are relayed to the cloud via a cellular modem onboard a Particle.io Boron device plugged into the control PC via USB. These data are then published to ThingSpeak.com, where they are graphed and easily accessible through any web-connected device.

### 2.2 Software

**Fig. 4:**
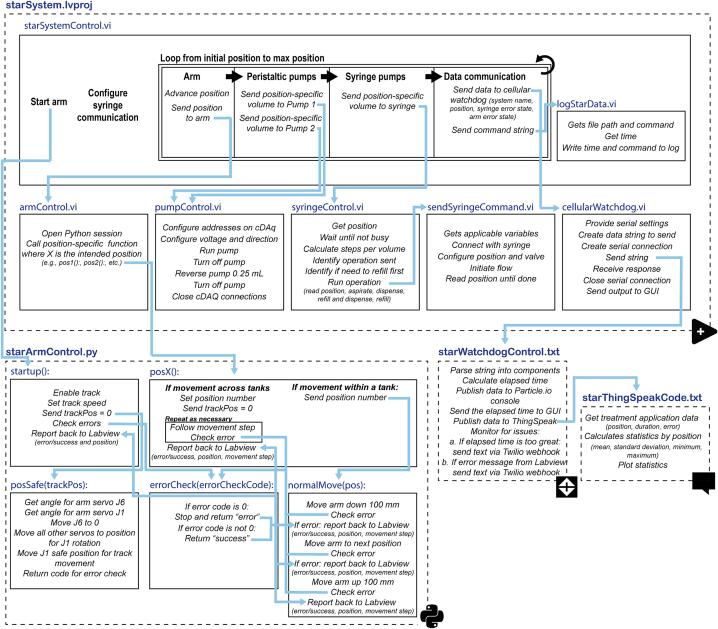
Graphical representation of the software logic and communication of the STAR system. Dashed boxes denote software languages and blue text refers to the various programs or Python functions, each outlined as a solid box. Individual steps are in italics and communication across programs/functions is illustrated with light blue arrow. (For interpretation of the references to colour in this figure legend, the reader is referred to the web version of this article.)

The STAR system and graphical user interface are programmed in LabVIEW (2021 SP1, Version 21.0.1, National Instruments). Control of the arm is achieved by calling Python functions for each movement step. The cellular watchdog device is programmed with the Particle IDE and ThingSpeak.com visualizations are programmed using Matlab (Fig. 4).

LabVIEW code - LabVIEW code includes eight different virtual instrument programs (VI’s) that are organized in a single LabVIEW project (starSystem.lvproj). Each VI is fully annotated, with descriptions of each operation step included in the code.

1. starSystemControl.vi - This VI comprises the main user interface and control program. The block diagram, where the program operates, has three separate components including 1. the initial startup, 2. a loop that controls the treatments and movements as the STAR system cycles through the beakers, and 3. a loop that receives settings/inputs and updates the graphical user interface. The control loop moves incrementally through beaker positions and contains a sequential structure, operating the robot arm’s movement, the peristaltic pumps, the syringe pump, and data communication (watchdog and logging), before advancing to the next position and repeating the process.
2. armControl.vi - This VI is called to control the robotic arm by calling Python functions and, in turn, receiving output strings and/or error codes.
3. cellularWatchdog.vi - This VI controls communication with the Particle Boron cellular watchdog device via USB.
4. globalVariables.vi - This file contains the global variables used by the VI’s.
5. pumpControl.vi - This VI receives a pump number (of two pumps) and required volume to dispense, along with pump-specific speed and flow rate, to direct the peristaltic pumps to appropriately dose. Following dosing, each pump reverses direction and aspirates a small volume to minimize dripping and cross contamination.
6. syringeControl.vi - This VI builds upon the Kloehn V6 multi-channel syringe instrument driver, available on the National Instruments instrument driver network. It receives the desired syringe operation and, depending on the current syringe position, sends commands to the Kloehn V6 (via the sendSyringeCommand.vi) to appropriately dispense, refill, or aspirate the syringe, along with the correct valve positions during each operation.
7. sendSyringeCommand.vi - This VI also builds on the Kloehn v6 instrument driver and controls the valve and syringe position.
8. logStarData.vi - This VI writes the appropriate data to the user-selected log file.

Python code - While the xArm can be run with the manufacturer’s included graphical user interface (xArm Studio), the STAR system employs the xArm Python Software Development Kit (SDK). Our code is fully annotated to assist with modification. Each arm has a unique IP address and multiple arms can be connected directly to a PC or local area network using network cables. A unique instance of this software is required for each STAR system, with the correct ARM-specific IP address and position coordinates in each. Upon startup, the arm checks for errors and initializes the linear track. It then moves the arm into a safe position, where the joints are sequentially adjusted to move the arm into a position occupying the smallest lateral radius (footprint). Following, the arm is rotated to be parallel with the axis of the linear track. These steps minimize the potential for encountering an obstacle should the robot boot up in an unexpected position and ultimately reduce the chance of impact during the linear rail transit.

Position coordinates can be input into the posArray for each of the 64 potential beaker locations. Each position includes cartesian coordinates (x,y,z, in mm), followed by a roll, pitch, yaw, and radius parameter. There are multiple solution paths by which the arm could traverse any two points. Since aquarium systems are complex and dynamic environments with numerous potential obstacles to avoid, we constrain movements to the treatment sequence and define each movement path as a separate step within each movement function. Each movement function is called by the LabVIEW code. If the movement is completed without encountering an issue, the function returns a statement of success along with the resulting position. If there is an error, it returns an “error,” along with the position and the individual movement step that led to the error within that movement function. These reporting and self-assessment steps help with debugging potential movement issues and can ensure proper system shutdown if the robot encounters an obstacle.

Movements between beakers within a single tank are executed by passing the intended position to the “normalMove” function, which raises the arm 100 mm along the z axis, laterally to the subsequent beaker, and then back down 100 mm to the injection position above the beaker. The first position within each tank (i.e., positions 1, 17, 33; 16 beakers per tank) is complicated by the additional steps required by the tank-to-tank transit. These transitions are unique and require an initial repositioning to the safe position, followed by a tank-to-tank transition that may require linear track movement. These individual movements are programmed separately rather than being handled with the “normalMove” function.

Stir plate code - The stir plate code is written in the Arduino IDE, and uploaded to Teensy (v3.5, PJRC) using the program Teensyduino. The stir plate program has two different modes: the “menu” mode where the user can edit each of the 16 motors’ voltages individually, and the “active” mode where the controller applies the user-input voltages to each motor. The GUI is displayed on an OLED screen, and the program is controlled with an infrared receiver and infrared remote control. The voltage settings input by the user are saved to a file on a micro-SD card on each PCB upon entering active mode, which allows for the settings to be saved between power cycles.

Cellular watchdog - The cellular watchdog is coded using Particle.io and ThingSpeak. It is not critical to the operation of the STAR system, though it is useful for quickly identifying and diagnosing potential issues. All code is extensively annotated. The Particle Boron board receives a comma delimited string from the LabVIEW code via serial communication (over USB). The code parses the string into the STAR System ID, position, a syringe error flag, and an arm error flag. The duration between receiving these strings is calculated, published on the Particle.io console, and then pushed to a ThingSpeak channel. Duration data are also sent back to LabVIEW. The Particle code monitors for issues including 1. if the duration between movements exceeds a threshold, and 2. if there are error flags. Should either of these scenarios occur, a text message is sent to the operator’s cell phone via a Twilio webhook. The ThingSpeak code calculates and plots descriptive statistics (mean, standard deviation, minimum, and maximum) for the duration the STAR system stays in each beaker position, based on the last 4500 data observations.

## 3 Design files summary

**Design files:** 

**Design file name**	**File type**	**License**	**Location**
starProgram.zip	LabVIEW code	CC BY 4.0	Source file repository
starArmControl.py	Python code	CC BY 4.0	Source file repository
starWatchdogCode.txt	Particle.io code	CC BY 4.0	Source file repository
starThingspeakCode.txt	Matlab code	CC BY 4.0	Source file repository
starStirCode.ino	Arduino code	CC BY 4.0	Source file repository
star3dPrintFiles.zip	STL files	CC BY 4.0	Source file repository
starLaserCutFiles.zip	Vector files	CC BY 4.0	Source file repository
stirControlBoard.zip	Eagle files	CC BY 4.0	Source file repository
starBillOfMaterials.xlsx	MS Excel file	CC BY 4.0	Source file repository

**starProgram.zip** is the LabVIEW code that runs the user interface, the peristaltic pumps, and the syringe pump. It calls the Python functions that control the arm position and relays information to the cellular watchdog. The starSystem.lvproj is the LabVIEW project file that contains the main program (virtual instrument, VI) named starSystemControl.vi and maintains all of the proper references among subvi’s.

**starArmControl.py** is the Python code for controlling the robotic arm movement.

**starWatchdogCode.txt** is the Particle.io code that is uploaded to the Boron cellular-enabled microcontroller.

**starThingspeakCode.txt** is the Matlab script that summarizes data sent from Particle.io to ThingSpeak.

**starStirCode.ino** is the Arduino code that runs the stir plate controller.

**star3dPrintFiles.zip** is a zipped folder that contains all of the CAD files for 3D printing the custom components of the STAR. It is organized into folders as follows: doserSystem (fluidRouting, pumpHousing), endEffector, stirSystem (sampleContainment, stirController, stirPlate), watchdog.

**starLaserCutFiles.zip** is a zipped folder that contains the vector files for laser cutting the custom acrylic components of the STAR. It includes two vector files with the parts arranged on them. Each is intended to be cut on a 24x24in piece of ¼in acrylic.

**starStirControlBoard.zip** is a zipped folder that contains the Eagle (Autodesk) board and schematic files used to create the stir controller circuit board.

## 4 Bill of materials summary

The detailed bill of materials is available as an Excel spreadsheet (starBillOfMaterials.xls) in the source file repository. It is organized by system and subsystem, where applicable. Costs are approximate and sources represent an option that was available in our purchasing systems at the time of development, rather than the authors' suggestion. It is likely that similar parts can be sourced for cheaper. Custom 3D printed parts are priced according to estimated material costs, as prices for fabrication can be highly variable.

## 5 Build instructions

Build instructions for the stir plate and doser system are provided as a pdf document (starBuildManual.pdf) in the source file repository. Additional details on connecting the STAR subsystems can be found in the hardware description and Fig. 3.

## 6 Operation instructions

**Fig. 5:**
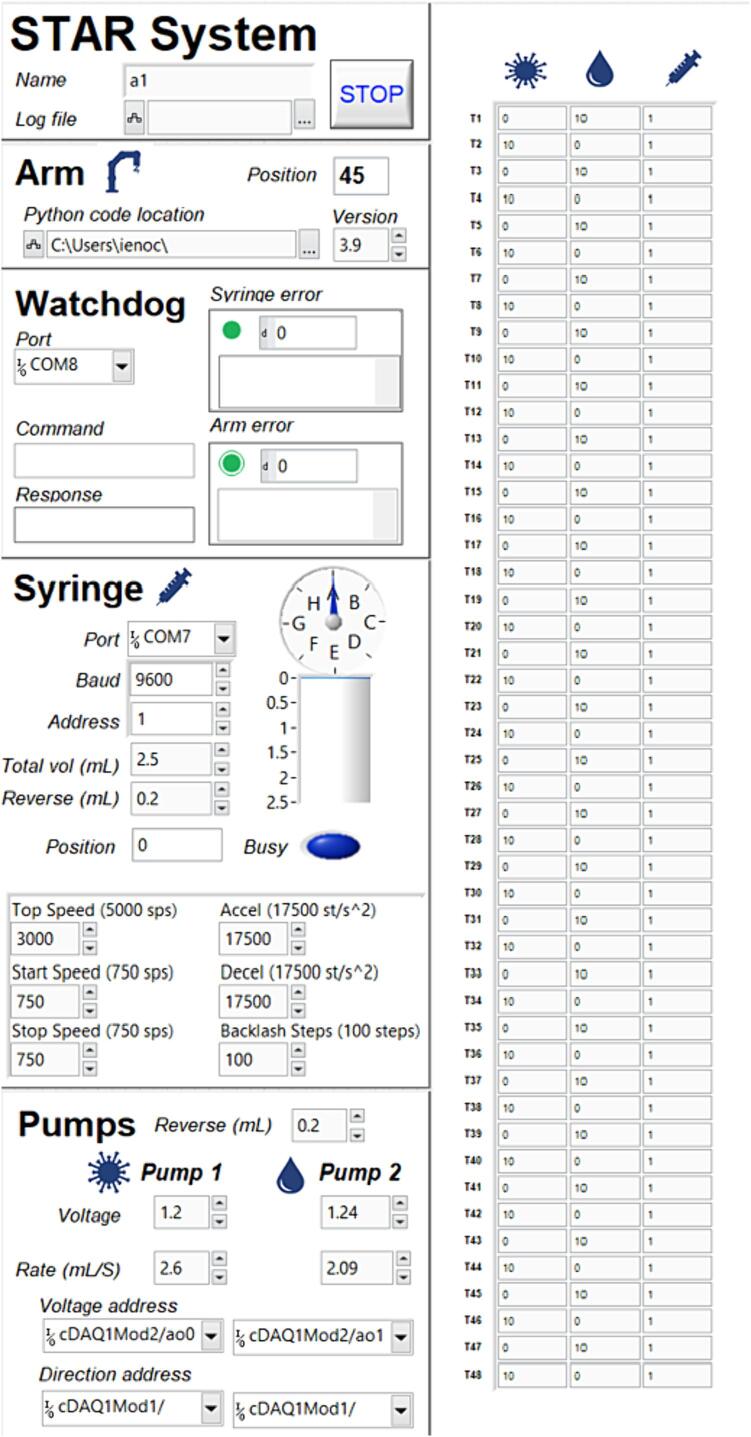
Graphical user interface of the STAR system, showing six subpanels that are used to control the system, arm, watchdog, syringe, pumps, and treatment levels.

The STAR system is controlled through the graphical user interface (GUI), the LabVIEW front panel of the starSystemControl.vi (Fig. 5). Multiple instances of the code may be used to control multiple systems simultaneously from the same PC. The GUI is arranged into six sub panels that control various aspects of the system's functionality as below. Settings can be saved as default within the front panel so that they do not need to be reentered after the program is restarted. In order to operate the robot control software, proceed through the sub panels in order as follows:

1. STAR System subpanel. Enter the name of the robot in the “Name” field, as well as the name and directory of the log file in the location provided. Stopping the program should be done through the stop button in this panel. Improperly shutting the program down with the LabVIEW abort execution button can lead to unintended system behavior.
2. Arm subpanel - This displays the current position (beaker) of the robot arm. Enter the location of the Python control code, as well as the version of Python being run in the fields as indicated.
3. Watchdog subpanel - This displays syringe and arm errors, as well as the control commands and responses from the arm-controlling Python code. Select the communication port on the PC that connects the Particle Boron watchdog device.
4. Syringe subpanel - This handles the control of the Kloehn V6 syringe pump. Enter the communication port, baud rate (9600), address (1), and the total volume of the syringe used (e.g., 2.5 mL). Also enter the reverse volume, the small volume aspirated immediately after dosing to minimize dripping. The volume is dependent on the length and type of tubing used and 0.2 mL was used during the testing of the system described herein. The position is the current location of the syringe within the 48,000 individual steps, which is translated to volume and displayed graphically, along with the current position of the valve above it. The “busy” light indicates when the syringe or rotary valve pump are undergoing an operation. The underlying user-selected parameters control the movement of the syringe. They should not need adjustment unless the syringe is having issues accurately dispensing fluid.
5. Pumps subsection - This subsection establishes communication between the two peristaltic pumps. The voltage field (0–3.3 V) controls the speed of the peristaltic pump and the rate of flow at that speed is entered into the flow field for each pump. Setting and calibration of these pumps is described in the “Pump calibration” section below. The voltage address for each pump refers to the slot location of the NI-9263 voltage control module in the CompactDAQ chassis, and the positions the wires are connected to on that module. Similarly, the direction addresses refer to the module slot and wire positions for the NI-9485 relay module that grounds the direction-control pins on the brushless motor controller. Selecting these addresses are handled in the drop down menus under each field. The address in each is parsed as follows: cDAQ number, which is 1 unless multiple cDAQ’s are used; Mod number, the module position in the cDAQ chassis from left to right; and channel address, the module-specific connections. For example, as seen in Fig. 5, the “Voltage address” is cDAQ1Mod2/ao0, which translates to the first (and only cDAQ) and the NI-9263 analog voltage module in position 2 of that cDAQ. The wire and position on that module is ao0, the first analog voltage position. More explanation can be found in the wiring diagrams of the build manual.
6. Treatment subsection - This subsection contains the volumes (mL) of each treatment to be applied to each beaker position. Enter the values into each field for each position as desired for the experiment you intend to run.

Pump calibration - Prior to running the STAR system, it is necessary to calibrate the peristaltic pumps and beaker positions. The speed of each pump is controlled by the voltage sent to each motor driver (0–3.3 V). Flow rates are dependent on that speed, but also several other parameters including the tubing diameter. It is recommended that the user first selects and inputs the voltage based on the fluid flow rates and behavior (e.g., splashing and dripping) desired. The pump/application-specific flow rate at that voltage should then be measured manually using a graduated cylinder and a stopwatch. That flow rate (mL s^−1^) should then be entered into the pump subsection under “Rate” for each pump. The flow rate is then used to calculate the correct duration the pump should run to achieve the beaker-specific dose volume as indicated in the treatment subsection. If flow rates are too low or high, voltage can be raised or lowered respectively, followed by re-measuring and adjusting the resulting flow rates.

Beaker position registration - Beaker positions are unique to each application. UFACTORY Studio (available at https://www.ufactory.cc) is a program that can be used to directly control the position of the xArm from the computer, or manually, while simultaneously outputting the corresponding cartesian coordinates. This program can be used to identify the positions of each beaker, which in turn should be entered into the posArray of the Python code. Detailed instructions for this step are provided in the build manual.

Running treatments - Once communication parameters and treatment volumes are appropriately entered into the front panel, and pumps and beaker positions are calibrated, press run on the starSystemControl VI. The system will continue to cycle through all positions, applying treatments as programmed.

## 7 Validation and characterization

Two STAR systems, each with 48 separate beakers, have been run continuously for a period greater than one month. During that time, they were successful at maintaining treatments, stirring the sample chambers, reporting data, and keeping coral alive. Additionally, testing was conducted to evaluate the potential for contamination, as well as the precision and accuracy of the STAR dosing system over time. Sixteen 600 mL beakers within the STAR system (see Fig. 1E) were continuously dosed with reverse osmosis (RO) water (30 mL) using one of the STAR peristaltic pumps. Additionally, yellow food-grade dye was added at four concentrations (0 %, 1.7 %, 3.3 %, and 5 %, n = 4 per treatment; McCormick & Company, Hunt Valley, MD) using the syringe pump, which added 0, 0.5, 1.0, and 1.5 mL of dye, respectively as programmed. Dye concentrations were measured using a UV/VIS spectrophotometer (Hach DR 6000, Loveland, CO) after creating a dilution curve to convert absorbance readings to percent concentrations.

Before the test began, initial target concentrations within each beaker were achieved by manually measuring the appropriate RO water and dye volumes using a 50 mL graduated cylinder and a 1000 μl pipette, respectively). The beakers were mixed for approximately five minutes (via stir plate), then dye concentrations were measured to confirm the target concentrations (represented as time 0 hrs). Following, the STAR system test was initiated with continuous automatic dosing at programmed levels. The beakers were dosed approximately every 11 min over a 36-hour period. Subsamples of treatment water were collected at 24 hrs and again at 36 hrs when the test was finally concluded. Kruskal-Wallis and post-hoc Dunn’s tests were used to compare the measured concentration of the four dye treatments at each time point.

**Fig. 6:**
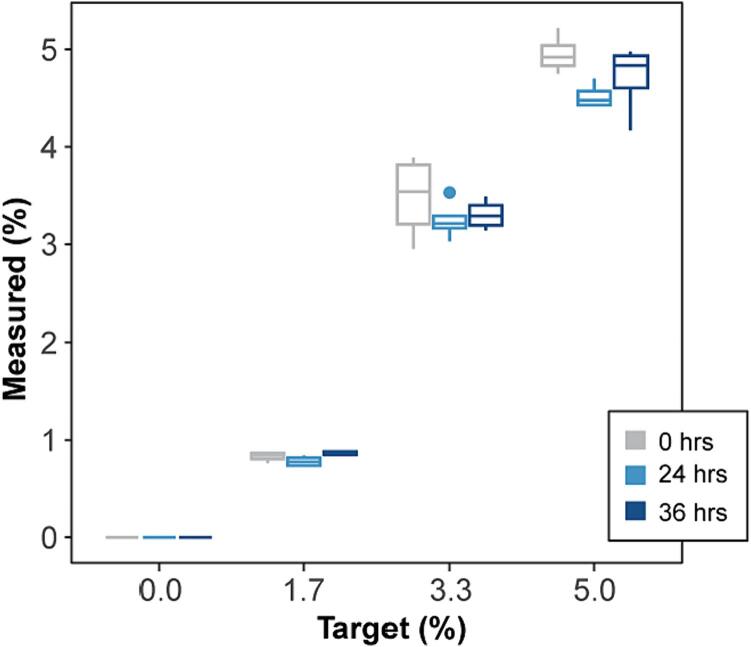
Measured concentrations (%) for each target concentration treatment over time. The 0 h collection was done on manually dosed treatments, while those at 24 and 36 h were produced by the STAR system.

The measured concentrations of the four treatments were significantly different from each other as intended (Fig. 6, p < 0.05). The measured concentrations within the 0 % target concentration beakers stayed at 0 %, suggesting that unintentional application of dye did not occur. Additionally, measured concentrations at each timepoint were not significantly different from each other (Fig. 6; 1.7 %, p = 0.07; 3.3 %, p = 0.66; 5 %, p = 0.08). This indicates that the STAR system dosed appropriately and maintained treatment concentrations over time.

### 7.1 Capabilities

• Robotic application of treatments to an array of independent beakers for high replication experimental studies on marine organisms
• High-precision dosing of nutrient solutions via a syringe pump
• High flow addition of two water sources via peristaltic pumps
• Treatment programming and calibration via a graphical user interface
• Maintenance of environmentally sensitive corals for periods greater than a month
• Error handling and performance assessment via a cellular connected device

### 7.2 Limitations and future directions

The STAR system described herein was built to investigate the impacts of nutrients, temperature, and SCTLD on coral in 600 mL beakers. There are, however, numerous additional applications and modifications that can be made to increase utility. The multi-position valve can, for example, be used to apply different treatment combinations. This includes, but is not limited to, designs that independently manipulate nitrogen and phosphorus, as nutrient ratios can be important drivers of organism responses to eutrophication [13].

The STAR system is also relevant to ocean acidification research, where sensitivity analysis is particularly important for predicting ecosystem persistence [14] and nonlinear responses are not uncommon [15]. Rather than exposing 48 organisms to high and low-CO_2_ treatments, the same number of organisms could be subjected to 48 different CO_2_ conditions, just by applying different ratios of high and low-CO_2_ water to each beaker with the peristaltic pumps. This would facilitate a regression-based design, which could provide information on non-linearity and tipping points, rather than simply whether an organism is responsive to a particular acidification scenario or not.

This type of sensitivity testing is particularly relevant to coral restoration research, where efforts are underway to evaluate the relative performance of different genotypes in order to make informed outplanting decisions [16]. Also pertaining to restoration and coral propagation, the STAR system could be leveraged to conduct automated targeted feeding, transferring suspended food media to the immediate vicinity of the coral polyps. This would potentially enhance growth and better prepare corals for environmental stressors [5], [17]. While it is outside of the immediate capabilities of the system presented here, the various subsystems of STAR could be modified to perform routine labor-intensive activities necessary for coral husbandry, including cleaning and algae control. This type of technology development is necessary for efficient coral propagation and reef restoration at scale.

There is also potential for modifying the STAR system for sampling and measurement, rather than (or in addition to) the treatment application presented here. Various sensors could be added to the end effector to collect data from individual beakers. If one of the beakers was designated as a clean water bath, a rinsing step could be added to the movement paths to minimize cross contamination. This approach would address potential financial constraints associated with the additive cost implications of instrumenting large numbers of treatment replicates.

## Ethics statements

Not applicable.

## CRediT authorship contribution statement

**I.C. Enochs:** Conceptualization, Funding acquisition, Methodology, Resources, Software, Supervision, Validation, Visualization, Writing – original draft, Writing – review & editing. **N. Soderberg:** Investigation, Methodology, Resources, Software, Validation, Visualization, Writing – original draft, Writing – review & editing. **A.M. Palacio-Castro:** Formal analysis, Funding acquisition, Investigation, Methodology, Resources, Supervision, Validation, Visualization, Writing – review & editing. **K. Eaton:** Formal analysis, Validation, Visualization, Writing – review & editing.

## Declaration of competing interest

The authors declare that they have no known competing financial interests or personal relationships that could have appeared to influence the work reported in this paper.
