# Supplementary material for: Sequential Treatment Application Robot (STAR) for high-replication marine experimentation
Source: HardwareX. 2024 Mar 28;18:e00524. doi: 10.1016/j.ohx.2024.e00524 (PMC11022082; doi:10.1016/j.ohx.2024.e00524)
Supplement: Supplementary data 1 [file mmc1.pdf]

Sequential Treatment Application Robot Subsystem Build Guide

Authored by Nash Soderberg, Ian Enochs, and Ana Palacio
Models and renderings by Ian Enochs and Nash Soderberg

Table of Contents

Table of Contents....1
1. Build Resources and Materials....1
3D Printed Components....1
Laser Cut Acrylic Components....2
Acrylic Gluing Best Practices....2
Tools and Bill of Materials....3
Systems and Organization....3
2. Robot System....5
Components....5
Preparing the Robot and Track....6
End Effector Assembly....6
Hose Guide Assembly....8
System Assembly and Plumbing....10
2. Dosing System and Control Hardware....11
Build Instructions....13
Power Distribution Assembly....13
Syringe Pump....15
Peristaltic Pumps....16
External Wiring and Computer Control....19
3. Stir System....22
Beaker Assembly....23
Beaker Rack....24
Acrylic Stir Plate Base Assembly....27
Stir Motor Assembly....29

Cable Router Assembly 31
Stir Plate Controller 32
Circuit Board Assembly 33
Controller Box Construction 35
Software Download and Setup 38
Arduino Libraries 38
Code Upload 40
MicroSD Installation 40
Software Usage 41
4. Cellular Watchdog 42
5. Computer Setup and Wiring 45
6. Robot Setup and Calibration 46
IP Address Configuration 46
One-time Calibrations 48
Position Calibration 50

Figures

Figure 1. Image showing systems and subsystems of STAR. 4
Figure 2. Robot system components. 5
Figure 3. End effector assembly. 7
Figure 4. Hose guide (top). 8
Figure 5. Hose guide (mid). 9
Figure 6. Hose guide (bottom). 10
Figure 7. Exploded CAD rendering of the doser housing design and photos. 13
Figure 8. Button wiring guide 14
Figure 9. Electrical connections to the Kloehn pump. 16
Figure 10. Motor wire harness and driver. 17
Figure 11. Diagram showing the wiring details of an individual peristaltic pump. 18
Figure 12. Images showing the peristaltic pumps wired to the drivers. 18
Figure 13. Image of a SP13 cable connector and bulkhead 19
Figure 14. Wire connections for two peristaltic pumps into the cDAQ's 21
Figure 15. Stir system assembly 22
Figure 16. Beaker assembly. 23
Figure 17. Sample stand. 24
Figure 18. Right beaker rack 25
Figure 19. Left beaker rack 26
Figure 20. Stir plate housing assembly 27
Figure 21. Assembly of the acrylic stir plate base 28
Figure 22. Assembly of the acrylic stir plate base 28

Figure 23. Stir system motor assembly 29
Figure 24. Fan motor modification. 30
Figure 25. Cable router assembly. 31
Figure 26. Photo of stir array 32
Figure 27. Stir plate controller circuit board 33
Figure 28. Stir system controller assembly 35
Figure 29. Photo showing potted cable routed to inside of the controller box 36
Figure 30. Controller box showing mounting holes for 2-56 threaded inserts 37
Figure 31. Photo showing the IR sensors, OLED screens, and push buttons 37
Figure 32. Images of the wires and connectors for the OLED screen and IR sensor 38
Figure 33. Image of the Teensy code upload window 40
Figure 34. Image showing details of the file to be saved to the micro SD card 41
Figure 35. Cellular watchdog assembly 42
Figure 36. Image of an antenna connected to the Particle Boron device. 43
Figure 37. Image of Particle's Web IDE 43
Figure 38. Image showing the main menu and Device tab of the Particle Web IDE 44
Figure 39. Photo of system control station. 45
Figure 40. Image of the Network and Sharing Center tab 47
Figure 41. Image of the Ethernet Status pop-up with the Properties tab 48
Figure 42. Image of the Ethernet Properties pop-up 48
Figure 43. Image of the IP Properties tab with an example IP address 49
Figure 44. Image of the opening page of the UFACTORY Studio software. 50
Figure 45. Image of the main menu of the UFACTORY Studio software. 50
Figure 46. Image of the STAR System python code to be edited 50
Figure 47. Image of the Linear Motor settings screen 51
Figure 48. Image highlighting the Friction Identification routine. 52
Figure 49. Image highlighting the collision sensitivity setting. 53
Figure 50. Image of the position array in the STAR System python code. 54
Figure 51. Image of the Live Control section of the UFACTORY Studio software. 55

Tables

Table 1. 3D printed components required for the construction of a STAR system. 1
Table 2. Kloehn SP13 wiring instructions. 15
Table 3. Corresponding wires of the CAT5 cable and DB9 to solder together. 20
Table 4. Arduino Libraries for Stir System Controller 39

1. Build Resources and Materials

These systems were designed to be accessible to those without any prior knowledge or experience with circuitry and electronics. However, familiarity with a few skills may be necessary for the construction and modification of this design such as soldering, 3D printing, laser cutting, and CAD software.

This build requires the use of some machinery that can be expensive to invest in, namely 3D printers and a laser cutter. Demand for this technology is high, however, and it is becoming increasingly available in local communities, or through vendors on the internet. If the builder is having trouble locating a source for any of these machines, they should investigate resources through universities and schools, public libraries, or local makerspaces.

If there are any difficulties with sourcing the exact materials or equipment listed in this build guide or the associated bill of materials, suitable alternative materials are commercially available and able to be used instead. The CAD designs and laser cut parts for these devices are all modifiable if the builder needs to retrofit them for their purposes.

3D Printed Components

The 3D printed parts that were used in this design were made in house. Parts were designed in Autodesk's CAD program Fusion 360, and were printed on a Formlabs Fuse 1 SLS 3D printer using Nylon 12 material. Although 3D printing is becoming increasingly available, if such access is unrealistic there are several companies where these parts may be ordered online.

Table 1. *3D printed components required for the construction of a STAR system, broken down into their systems and subsystems where applicable.*

System	Subsystem	Component	File Name	Quantity	Figure
cellular watchdog	cellular watchdog	watchdog housing	watchdogHousing.stl	1	39.1
		watchdog lid	watchdogLid.stl	1	39.5
doser system	pump housing	doser housing	doserHousing.stl	1	7.16
		doser vent side	doserVentSide.stl	1	7.18
		doser pump mount	doserPumpMount.stl	1	7.1
robot system	end effector	end effector	endEffector.stl	1	3.8
	fluid routing	top hose guide A	topGuideA.stl	1	4.1
		top hose guide B	topGuideB.stl	1	4.1

		mid hose guide A	middleGuideA.stl	1	5.1
		mid hose guide B	middleGuideB.stl	1	5.1
		bottom hose guide A	bottomGuideA.slt	1	6.1
		bottom hose guide B	bottomGuideB.stl	1	6.1
stir system	beaker assembly	injection snorkel	injectionSnorkel.stl	16	16.1
	stir controller	stir controller box	stirControlBox.stl	1	28.9
		stir controller lid	stirControlLid.stl	1	28.2
		stir controller port screw	stirControlClip.stl	2	28.17
		stir controller clip	stirControlScrew.stl	2	28.10
	stir plate	short mounting arm	armShort.stl	2	20.7
		long mounting arm	armLong.stl	2	20.4
		fan motor base	fanMotorBase.stl	16	23.4
		magnet holder	magnetHolder.stl	16	23.2
		wire routing hook	wireHook.stl	12	25.1

Laser Cut Acrylic Components

This design utilizes laser-cut quarter inch acrylic to build the housing for the stir plate array, as well as to protect the screens for the controller box. The acrylic used for this project was purchased as 24 inch by 24 inch sheets, and it is possible to cut all necessary parts for one stir plate system out of two sheets of this size, with some unused area of the second sheet left over. The laser used for the original build is a Boss LS-2436 150W. If the builder does not have access to a laser cutter, a CNC could also be used to cut from stock acrylic, or the pieces can be ordered from an online vendor. Please note that the doser box does not require any laser cut components. ***If the builder is cutting from 24 inch by 24 inch sheets of quarter inch acrylic, files compiled with all of the necessary parts efficiently laid out have been provided***.

Acrylic Gluing Best Practices

Several of these acrylic parts will need to be glued together through the use of acrylic adhesive. As a general workflow for using acrylic adhesive, wear PPE to protect exposed skin and eyes. On a clean, flat work surface, lay out spare paper or cardboard to protect the work area from excess acrylic adhesive. Place the parts to be glued on the work surface, and use painters tape

to position the parts to the appropriate orientation. Using a syringe, selectively apply acrylic adhesive to areas that should be glued. Follow the instructions on the adhesive container to determine how long the acrylic needs to cure for a working bond.

Tools and Bill of Materials

This project is accompanied by a bill of materials for all the electronics, hardware, and consumables required for the construction of a STAR system. The bill of materials may be found in the source file repository (https://zenodo.org/doi/10.5281/zenodo.10048123). Some of the necessary and recommended tools for construction are as follows: laser cutter, 3D printer, soldering iron and associated equipment and supplies, screwdrivers (Phillips and flathead), nut drivers (7/16”, 1/4”), masking tape or painters tape, 30 gauge ½” syringe, flush cutters, wire stripper, heat gun, and a ¼-20 tap.

Systems and Organization

For ease of use, this document is organized into the same systems and subsystem components referenced in the manuscript including the robot system (tube routing guides and end effector), dosing system, stir system, and cellular watchdog. Systems with multiple subsystems are organized into subfolders inside the parent system. This same organizational scheme is also used in the source file repository.

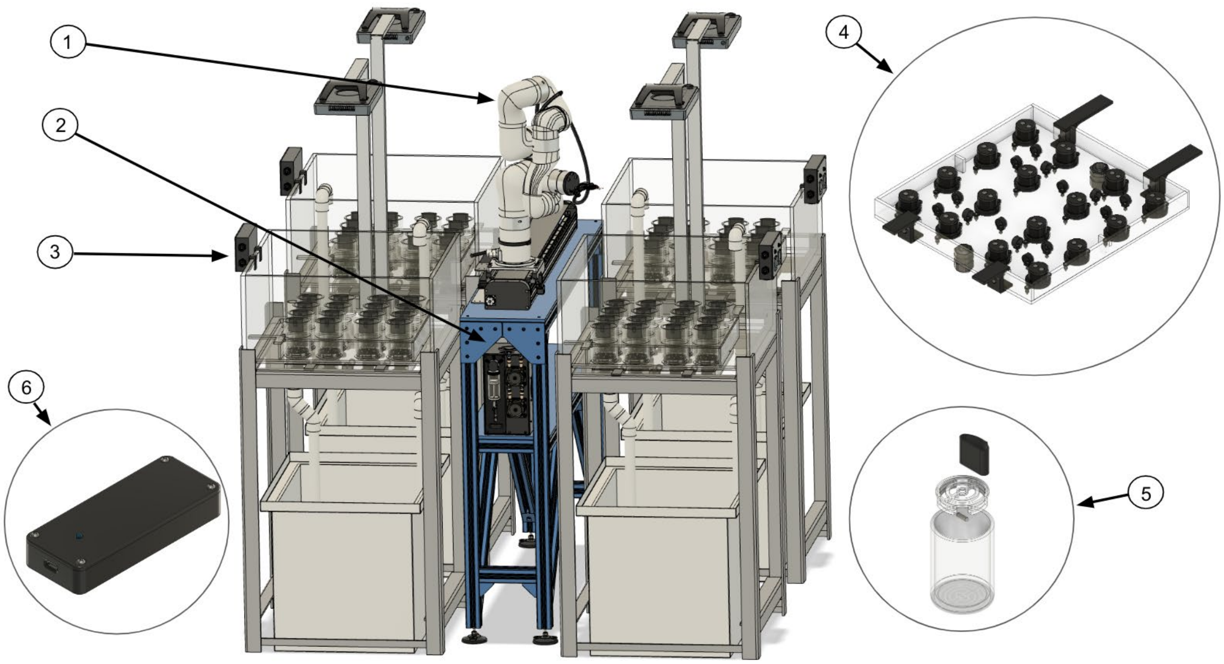

Figure 1. Image showing systems and subsystems of STAR including the **(1)** robot system consisting of a UFACTORY, a linear motor, a 3D printed end effector, and a series of 3D printed hose guides to run tubing along the length of the arm and linear motor to the **(2)** dosing system made from two peristaltic pumps and a Kloehn syringe pump inside a custom 3D printed housing. A **(3)** controller for a custom **(4)** stir system mounted under the aquariums keeps the water inside the **(5)** beaker assemblies from stagnating. A **(6)** cellular watchdog monitors activity on the STAR system computer.

2. Robot System

Components

The robot component of the STAR system consists of a robotic arm (Figure 2.1) mounted on a linear motor (Figure 2.5) using six M5 screws (Figure 2.6), provided with the robotic arm and linear motor, to service four separate aquaria. Three lines of tubing (Figure 2.4) are led from the dosing system, along the side of the linear motor's cable guide, up the robot arm all the way to its end effector (Figure 2.8) from which it dispenses the liquids from the dosing system into the research vessels. A series of hose guides (Figure 2.2, 2.3, 2.7) organize the tubing along the length of the robot arm and keep the robot from getting tangled.

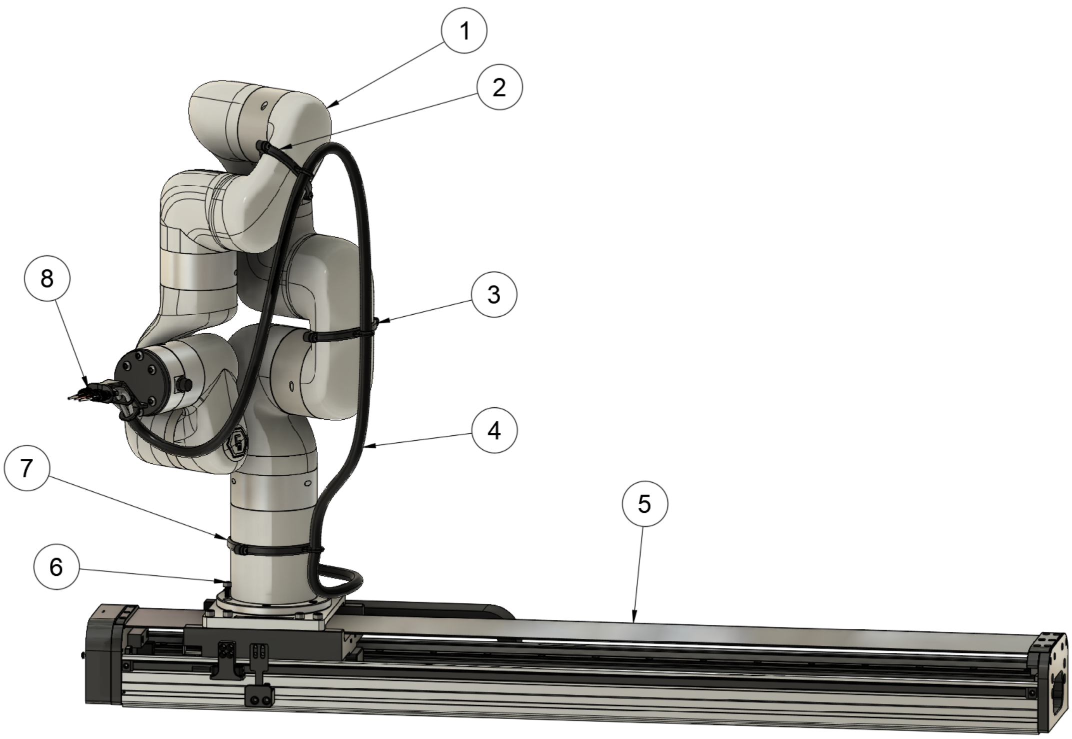

Figure 2. Robot system components including **(1)** xArm 6, **(2)** hose guide (top), **(3)** hose guide (mid), **(4)** tubing, **(5)** linear motor, **(6)** screw (6 count(ct), M5, 12 mm long, stainless steel), **(7)** hose guide (bottom), and **(8)** end effector.

Preparing the Robot and Track

To protect the robot arm and linear track from potential salt or water damage, wrap the robot and track in 2” electrical tape. While taping, make certain any joints on the robot or points of movement on the linear motor are not covered or restrained. If any joints on the robot arm do get covered in tape, use a razor blade to carefully slice along the joint and free up the robot for movement.

Mount the linear track (Figure 2.5) on the desired platform with hardware that is appropriate for the thickness of the platform the track is being installed on, and secure the robot arm (Figure 2.1) to the linear track (Figure 2.5) using the hardware provided by Ufactory (Figure 2.6). The robot must be mounted on a sturdy platform to ensure the robot does not cause the platform to tip or wobble.

End Effector Assembly

To install the 3D printed end effector (Figure 2.8) on the end of the robot arm (Figure 2.1), use six M6 screws (Figure 3.7) to screw the end effector into place. There is a small cylinder on the end effector that will mate with a small indentation on the end of the robot arm, so only one mounting orientation is possible.

Twist three threaded Luer lock to ⅛” barb connectors (Figure 3.5) into the end of the end effector until they are fully threaded on. To each of those, twist on a male to male Luer lock connector (Figure 3.4).

The end effector for the robot is equipped with three separate dispensing tips (Figure 3.1, 3.2, 3.3) which can be swapped out with tips of various gauges to meet the researcher’s needs. In Figure 3, two wider gauges are installed on the sides of the end effector which are plumbed to the peristaltic pumps of the dosing system. A smaller gauge tip in the center of the end effector is connected to the syringe pump which provides lower flow rates and volumes, with a lower likelihood of dripping.

While the peristaltic pumps use ⅛” inner diameter (ID) black airline tubing (Figure 3.6) for fluid routing, the syringe pump uses a smaller 1/16” ID line of tubing due to smaller volume needs. This 1/16” ID tubing will not be able to interface with the ⅛” barb to Luer connector (Figure 3.5), so use a small length of ⅛” black tubing to interface with the barb to Luer connector and then install a ⅛” to 1/16” barb reduction connector (Figure 3.9) to make the assembly compatible with the 1/16” tubing.

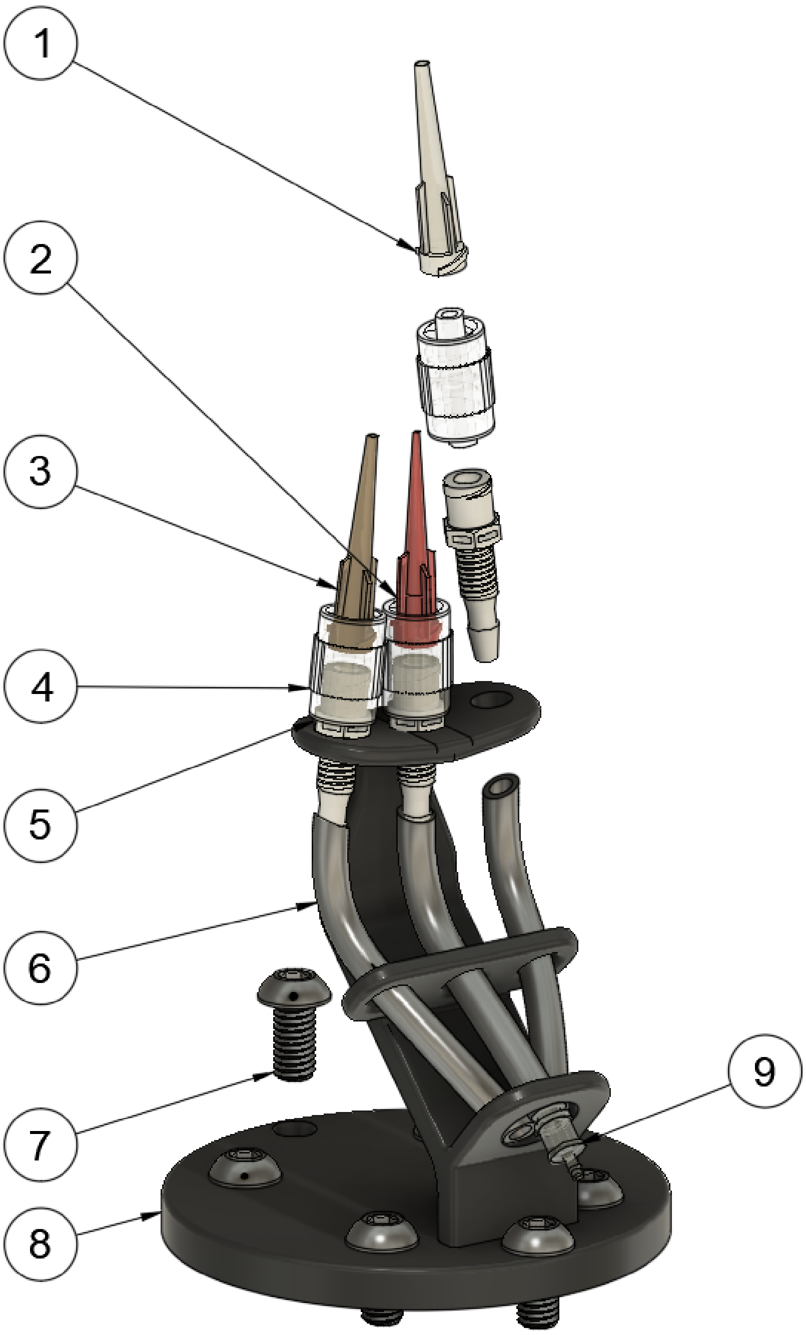

Figure 3. End effector including **(1)** 14 gauge dispensing tip (white), **(2)** 25 gauge dispensing tip (red), **(3)** 16 gauge dispensing tip (brown), **(4)** Luer lock connector (3 ct, male to male), **(5)** Luer lock connector (3 ct, female luer lock to barbed 1/8” ID tubing, ¼-28 thread), **(6)** tubing (1/8” ID), **(7)** screw (6 ct, M6, 12mm long, stainless steel), **(8)** 3D printed end effector, **(9)** ⅛” to 1/16” ID barbed reduction.

Hose Guide Assembly

Lengths of tubing are run from the dosing system (Figure 1.5) to the end effector of the robot (Figure 2.8), and are routed through three hose guides spaced periodically along the robot arm (Figure 2.2, 2.3, 2.7). The top (Figure 4), mid (Figure 5), and bottom (Figure 6) hose guides are of different sizes and shapes to fit the contours of the robotic arm (Figure 2.1) . They are, however, each assembled in the same manner. Each contains two 3D printed half collars (Figure 4.1, 5.1, 6.1), that are held together using 6-32 ½" stainless steel screws (Figure 4.2, 5.2, 6.2). Press the 6-32 threaded brass inserts (Figure 4.3, 5.3, 6.3) into the receiving end of the hose guide assemblies as shown in Figures 4-6, and then screw them in place on the robot arm as shown in Figure 2.

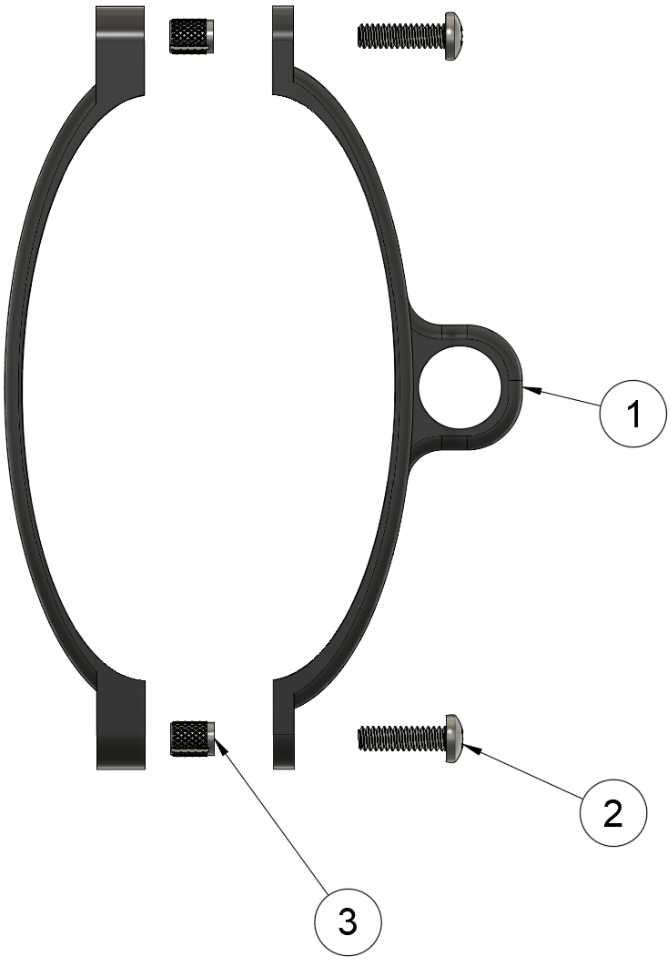

Figure 4. Hose guide (top) including **(1)** 3D printed hose guide pieces, **(2)** screw (2 ct, 6-32, ½" long, stainless steel), **(3)** threaded insert for plastic (2 ct, 6-32, ¼" long, brass).

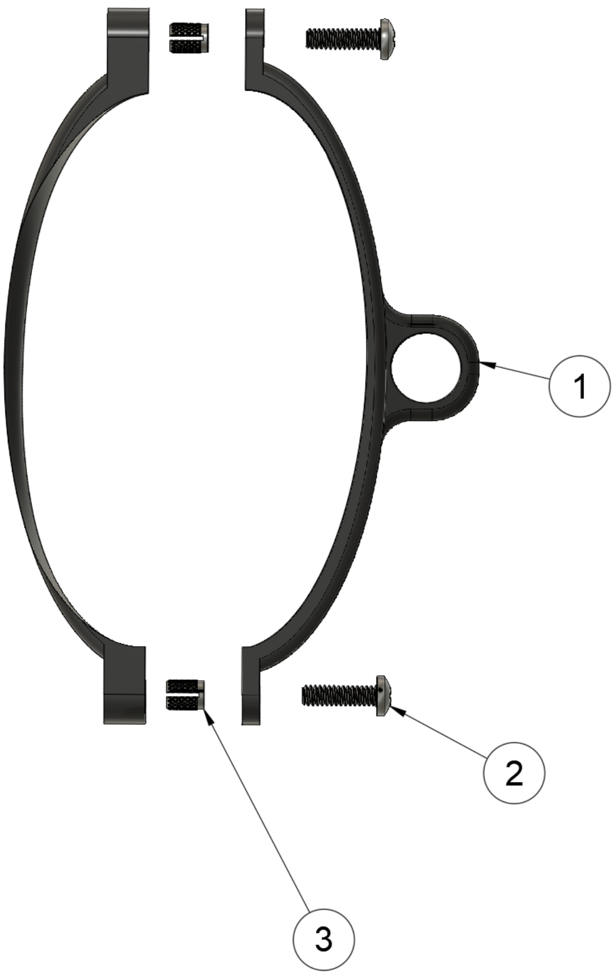

Figure 5. Hose guide (mid) including **(1)** 3D printed hose guide pieces, **(2)** screw (2 ct, 6-32, ½" long, stainless steel), **(3)** threaded insert for plastic (2 ct, 6-32, ¼" long, brass).

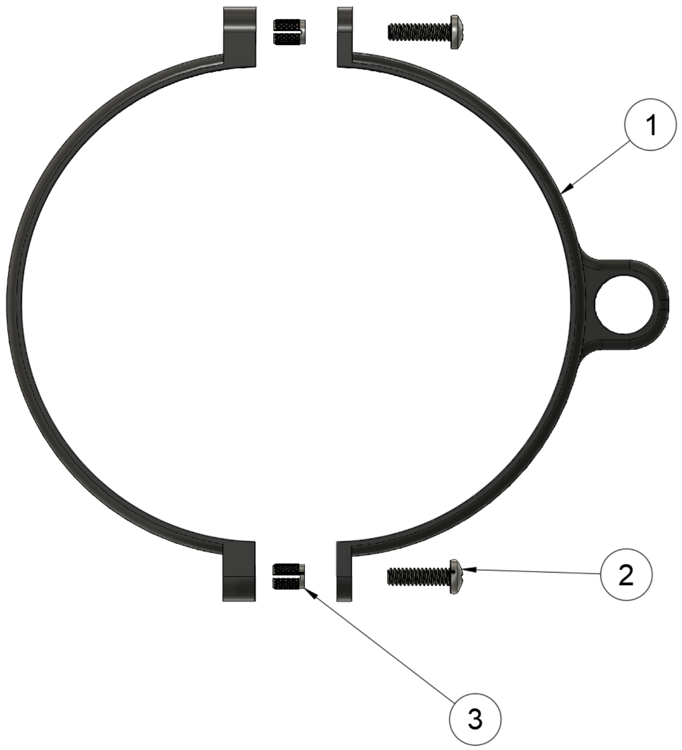

Figure 6. Hose guide (bottom) including **(1)** 3D printed hose guide pieces, **(2)** screw (2 ct, 6-32, ½” long, stainless steel), **(3)** threaded insert for plastic (2 ct, 6-32, ¼” long, brass).

The tubing must be prepared prior to being threaded through the hose guides. Cut three separate ten foot lengths of black airline tubing. Two of these lengths will be used as-is to connect the peristaltic pumps of the dosing system to the end effector. With the third length of tubing, use a razor blade to cut along the length of the tubing so there is a slit that goes from one end of the tube to the other.

Cut a ten foot length of clear Tygon tubing. Peel apart the black airline tubing that was cut in the last step, and nest the length of Tygon tubing inside of the airline tubing. This acts as a step of protection against biofouling as well as bending of the thinner Tygon tubing which will be connected to the syringe pump of the dosing system.

System Assembly and Plumbing

With all three of these prepared tubing lines, insert them into ¼” expandable sleeving. Do not pre-cut the sleeving as the exact length of sleeving that will be required will depend on how expanded the sleeving itself is. Instead, once the tubing is mostly collected inside the sleeving sans a few inches of tubing at both ends to allow for connections, cut the sleeving at the appropriate length.

The resulting hose, consisting of the three lengths of tube collected inside of the expandable sleeving, can be routed along the cord track for the linear motor of the robot, threaded through the holes of the hose guides (Figure 4.1, 5.1 and 6.1), and ultimately connected to the barbed connectors of the end effector assembly. Using zip ties or other sturdy cordage, attach the tail end of the hose along the length of the cord track of the linear motor. Doing this ensures that the hose will remain out of the way of the robot should it move on the linear track. The end of these tubes will be connected to the dosing system when that is constructed.

2. Dosing System and Control Hardware

The STAR system doser box (Figure 7) contains two calibrated peristaltic pumps (Figures 7.3, 7.4) for dosing liquids at a high rate (up to 690 mL min^{-1}), as well as a Kloehn syringe pump (Figure 7.19, 8.19) for high precision and accuracy dosing. The peristaltic pumps are driven by brushless DC motors for continuous-use, and require motor drivers (Figure 7.24). The drivers are mounted on the interior of the box on standoffs (Figures 7.22) using 4-40 screws (Figure 7.23), as well as on the exterior of the doser box to mount the standoffs. A female panel mount barrel jack for 24V DC power (Figure 7.15), two SP13 connectors for pump communication (Figure 7.10, 7.11), and an LED push button to toggle power on and off (Figure 7.17) are located on the back of the box. A 24V fan mounted on the inside of the box cools the system (Figure 7.9). Power for the system is distributed from the push button using a power distribution board (Figure 7.14). There are three 3D printed parts needed for the construction of this device: the doser box (Figure 7.16), doser box vent (Figure 7.18), and pump mount panel (Figure 7.1). Only one of each is needed.

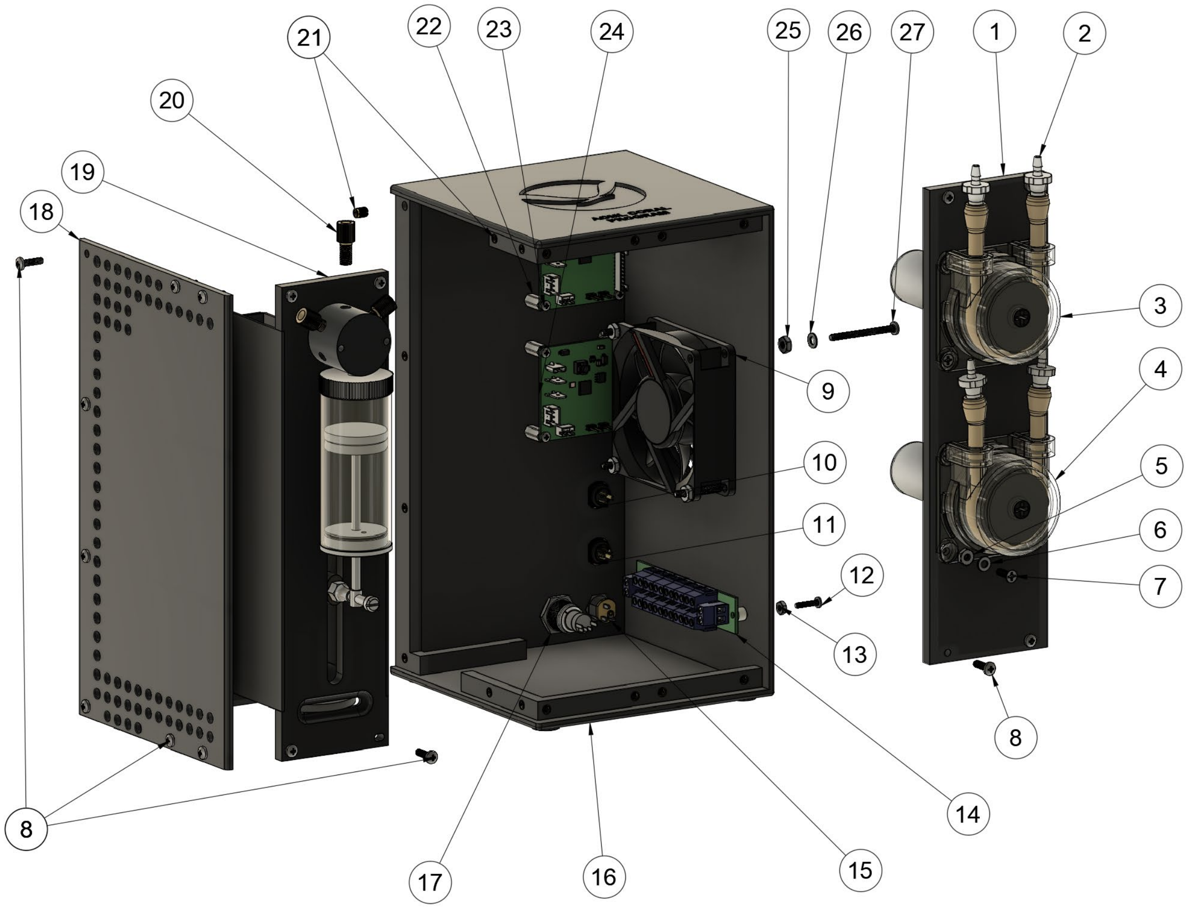

21
22
23
24
25
26
27
1
2
20
19
18
3
9
4
10
5
11
6
12
7
13
8
14
8
17
16
15

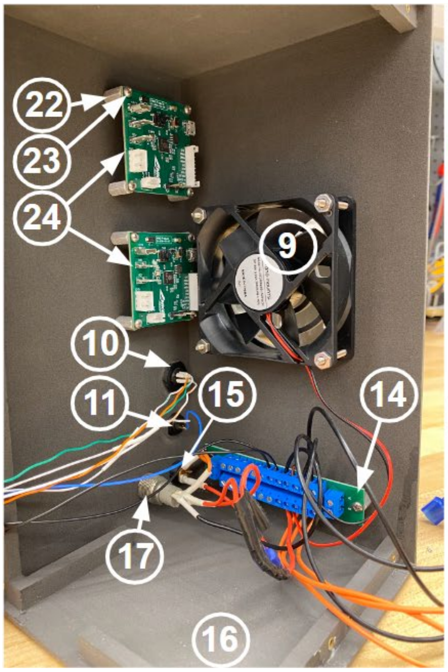

22
23
24
9
10
11
15
14
17
16

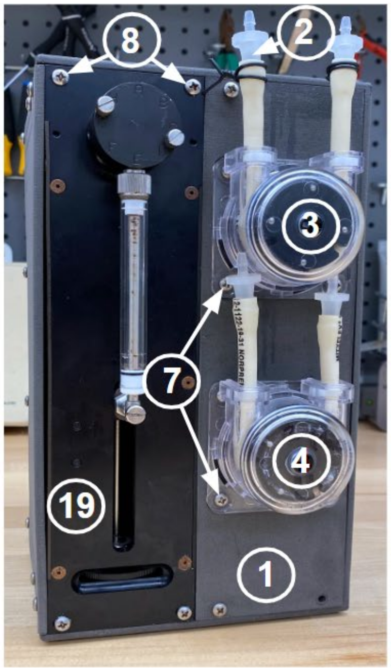

8
2
3
7
4
19
1

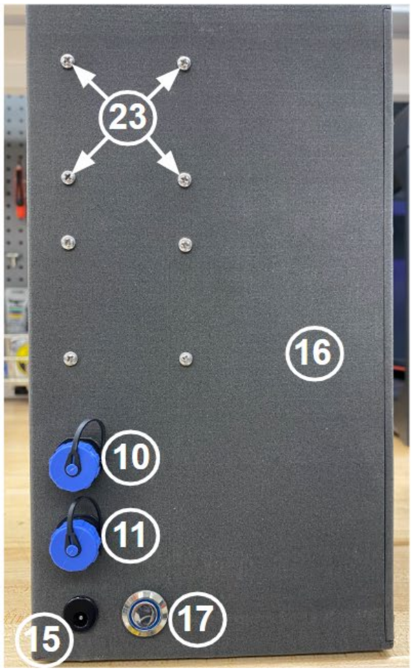

23
16
10
11
15
17

Figure 7. An exploded CAD rendering of the doser housing design (top) and photos (bottom) showing a fully constructed assembly including **(1)** 3D printed doser pump mount, **(2)** barb fitting (1/8” to 1/4” ID), **(3)** peristaltic pump (top), **(4)** peristaltic pump (bottom), **(5)** hex nut (8ct, 6-32, stainless steel), **(6)** washer (8ct, #6, stainless steel), **(7)** screw (8 ct, 6-32, ½” long, stainless steel), **(8)** screw (16 ct, 6-32, ½” long, stainless steel), **(9)** computer fan (80mm, 24v), **(10)** bulkhead connector (sp13, 4 pin), **(11)** bulkhead connector (sp13, 3 pin), **(12)** screw (2 ct, 4-40, ½” long, stainless steel), **(13)** hex nut (2ct, 4-40, stainless steel), **(14)** power distribution board, **(15)** barrel jack (DC), **(16)** 3D printed doser housing, **(17)** metal pushbutton, **(18)** 3D printed doser hosing side vent, **(19)** Kloehn syringe pump, **(20)** Supelco ⅛” flangeless fitting, **(21)** threaded insert for plastic (16 ct, 6-32, ¼” long, brass), **(22)** threaded female standoff (8 ct, ¼” hex, 4-40, ½” long, aluminum), **(23)** screw (16 ct, 4-40, ¼” long, stainless steel), **(24)** motor controller (2 ct). Top image is a model of the doser box, bottom images are real photos of a doser box being constructed. Numbers correspond to both sets of images.

Build Instructions

A diagram showing the connection of the doser box, pumps, and cDAQ to the computer can be found in the main manuscript (Figure 3). Build instructions are organized below into assembly power, syringe pump, peristaltic pumps, and external wiring and cables.

Power Distribution Assembly

To begin, install the power distribution board (Figure 7.14) on the inside of the 3D printed doser housing (Figure 7.16) using a ½” 4-40 screw (Figure 7.12), the header included with the power distribution board, and a 4-40 hex nut (Figure 7.13). The doser box requires 24V DC power input into the panel mount barrel jack (Figure 7.15). On the panel mount DC barrel jack (Figure 7.15), solder the positive (red, 18 AWG) and negative (black, 18 AWG) wires to the corresponding pins. The positive wire will be terminated in a female quick disconnect connector, which will need to be crimped to some exposed wire. The negative wire will lead to the negative terminal on the end of the power distribution board.

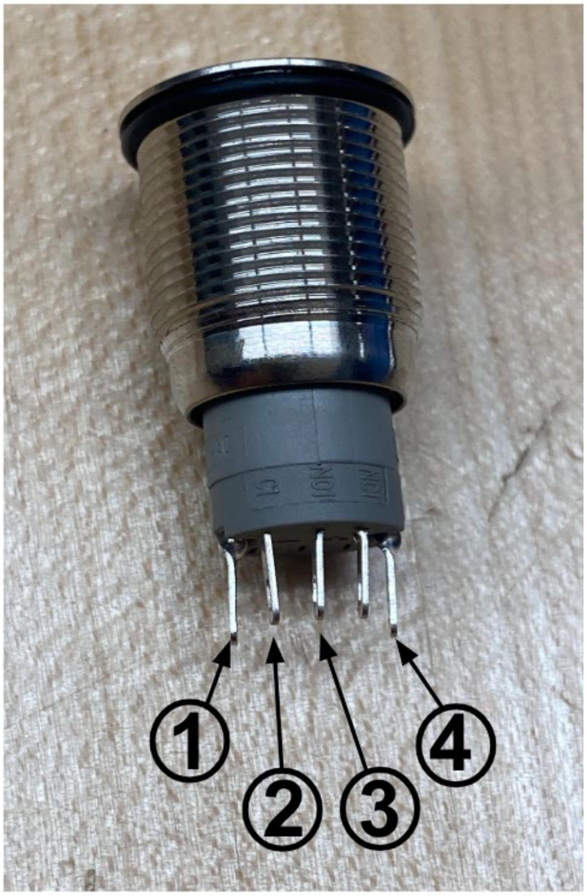

Figure 8. Button wiring guide showing **(1)** ground, **(2)** power in (24v DC), **(3)** power out, **(4)** LED power

On the metal pushbutton (Figures 7.17, 8), solder another small length of red 18 AWG wire to the power pin (Figure 8.2) and terminate the wire in a male quick disconnect connector, crimped to exposed wire. This wire should easily connect and disconnect to the quick disconnect fitting crimped to the positive wire on the barrel connector. This quick disconnect allows for easier maintenance if either the barrel jack or the power button need to be replaced. To the power out pin (Figure 8.3), solder a length of red 18 gauge wire and route it to the power terminal at the end of the power distribution board. To the ground pin on the metal pushbutton (Figure 8.1), solder a length of black 18 AWG wire. The other end of this wire will be screwed into one of the ground terminals on the power distribution board. Lastly, to the LED power pin (Figure 8.4) solder a length of 18 gauge red wire with a 470 Ω resistor in-line with the wire and screw the end into one of the screw terminals on the power side of the power board. This resistor is necessary to bring voltage down to appropriate levels for the LED light in the pushbutton. Without it, the LED will overheat and burn out. Figure 7 shows where each part should be installed on the doser box housing (Figure 7.16).

When the assembly has a 24V power source attached to the barrel jack and the push button is not activated, the assembly remains grounded but no power is delivered to the power board and

the system remains off. When the push button is activated, power is allowed to pass from the button to the power board which, in turn, lights up the LED indicator light in the button and powers all other electronics in the doser box.

Syringe Pump

To begin the installation of the syringe pump, first install the card edge adaptor as seen in Figure 9 to the exposed circuit board edge on the back of the Kloehn pump (Figure 7.19).

Communication with the Kloehn syringe pump is handled through RS232 serial communication out of the three pin SP13 connector on the doser box (Figure 7.11). Cut a length of CAT5 cable around six inches and then remove the eight individual wires from the outer sheath of the cable. The brown, blue, and blue striped wires will be used to connect the SP13 connector to the card edge adaptor. Each pin on both the bulkhead and cable end of the SP13 connector will be numbered, where matching numbered pins connect each other between the two pieces. Table 2 shows what wires should be soldered to which pin on the SP13 bulkhead. Once the appropriate wires have been soldered to the appropriate pins, the non-soldered ends should be stripped, crimped with Molex KK crimps and fit into the appropriate segment on a 3-pin female Molex KK connector, so when it is plugged into the RS232 section of the Kloehn pump the wires for each function are routed to the respective pin (Figure 9.1). Attach the Molex KK connector to the card edge adaptor.

Table 2. *Kloehn SP13 wiring instructions showing wires to solder to which pin of the SP13 connector for Kloehn pump communication.*

Function	Wire Color	SP13 Pin Number
RXD - Receive data	Blue striped	1
GND - Ground	Brown	2
TXD - Transmit data	Blue	3

The 24V power from the power board also needs to be routed to the Kloehn pump. Using 18 gauge black and red wires for ground and positive respectively, screw one end of each wire into a terminal on their respective side of the power board. On the other end, attach a Molex KK crimp. Utilizing the top two pins for power on the card edge adaptor (Figure 9.2), route the wires into their respective pins on a four pin female Molex KK connector. Attach to the card edge adaptor.

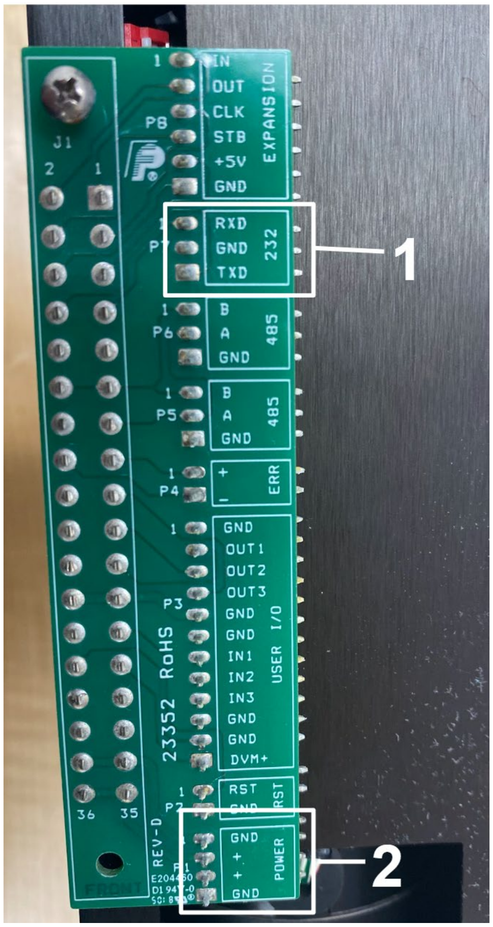

Figure 9. Electrical connections to the Kloehn pump including **(1)** RS232 (TX, RX, and ground pins) connected to the bulkhead connector (SP13, 3pin, Figure 6.10), **(2)** power (24V DC, and ground) connected to the power board (Figure 6.14).

Peristaltic Pumps

The other SP13 connector, with 4 pins, is for the peristaltic pumps on the doser box. Two communication wires from this SP13 connector are required for each peristaltic pump driver: one that delivers signal voltage which determines the speed of the motor using pulse width modulation or PWM (Figures 10A.4, 10B.4), and another wire which will determine the direction the motor turns (Figure 10A.3). The motor driver also requires access to 24V power from the power board (Figure 10B.5) and grounding (Figure 10B.6). Three different kinds of wire harnesses are necessary for construction of this assembly: the 6 pin motor wire harness that plugs into the motor of the peristaltic pump (Figure 10A), a three pin wire harness for 24V power and PWM (Figure 10B.1, 10B.2), and a smaller three pin wire harness for potentiometer signal which will come from the SP13 connector and another pin for ground (Figure 10B.7, 10B.8 right). The third pin will remain unused in this last harness.

To get started with wiring the peristaltic pumps, unpackage the motors and their drivers. Inside the packaging of the drivers there should be a collection of wires and harnesses preconfigured

for various use cases. None of the preconfigured harnesses perfectly match this use case, so some modifications must be made.

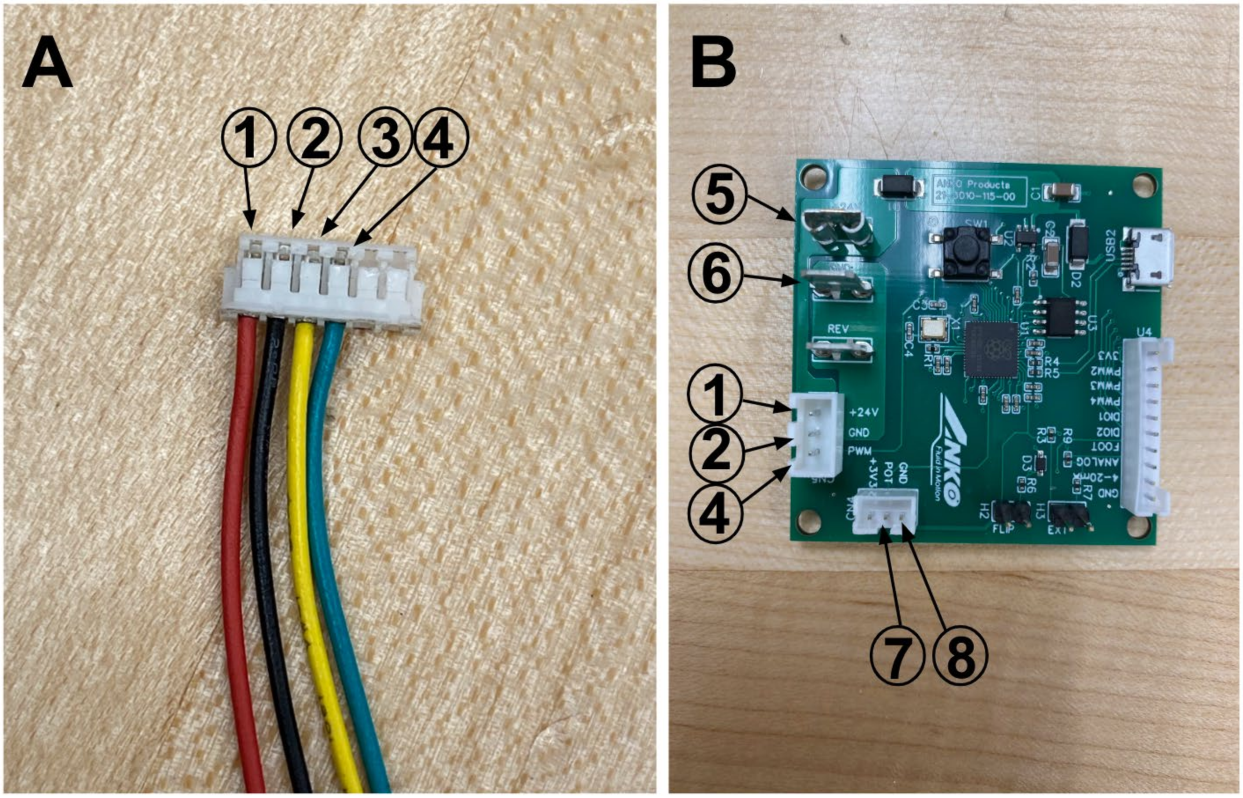

Figure 10. Motor wire harness **(A)** and driver **(B)** showing **(1)** 24V DC to motor, **(2)** ground to motor, **(3)** direction pin to bulkhead connector (SP13, 4pin, Figure 7.11), **(4)** PWM pin, **(5)** 24V DC, **(6)** ground, **(7)** motor speed (0-3.3V DC) to bulkhead connector (SP13, 4pin, Figure 7.11), **(8)** ground.

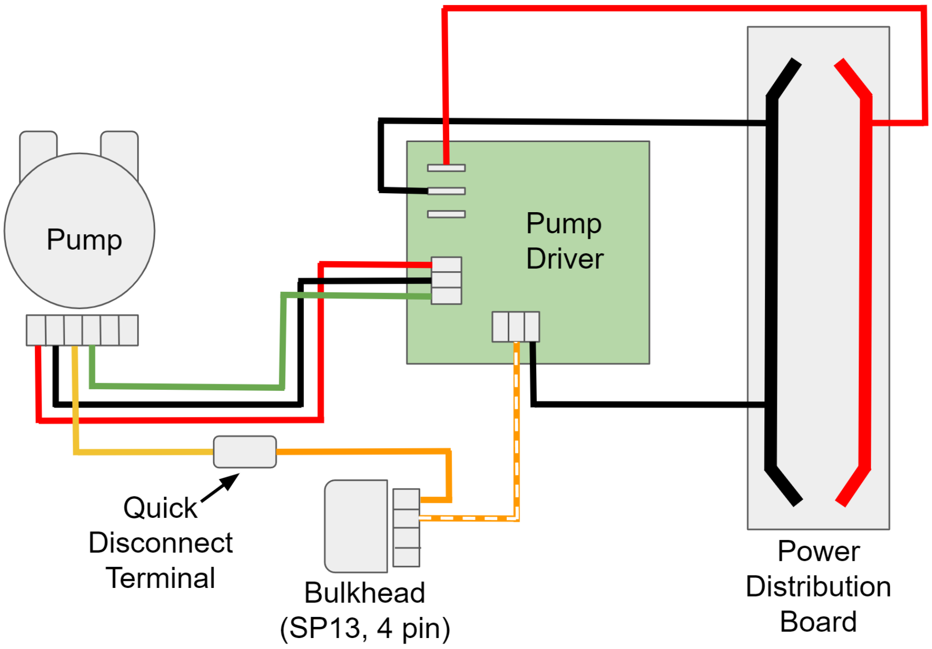

Figure 11. Diagram showing the wiring details of an individual peristaltic pump.

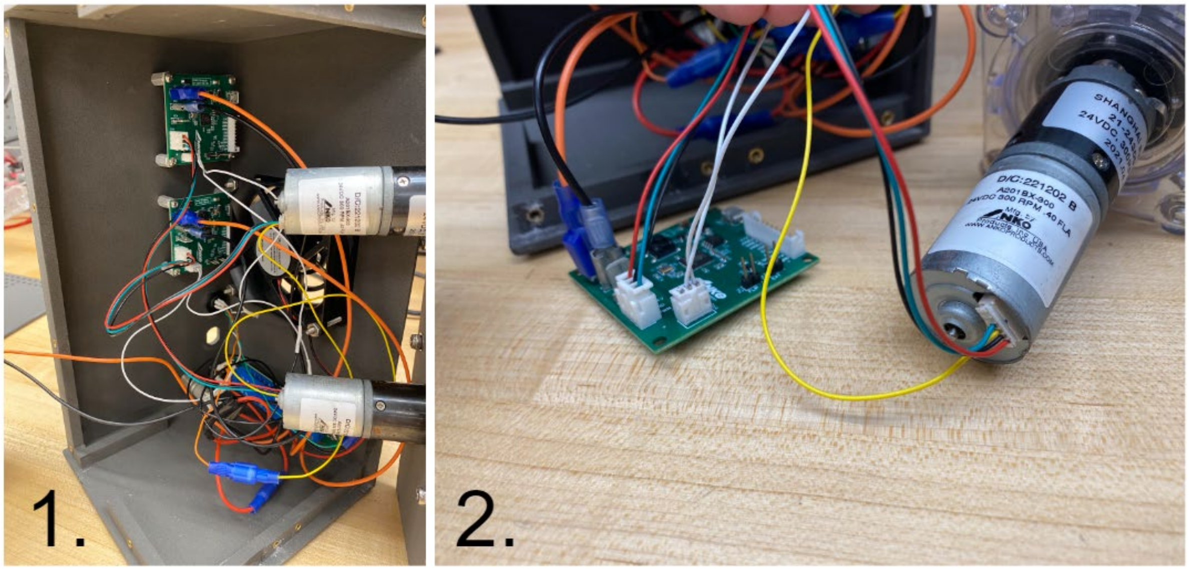

Figure 12. Images showing **(1)** the peristaltic pumps wired to the drivers, which are installed on the doser housing, and **(2)** closer look at details of the wire harnesses on the peristaltic pumps and drivers

External Wiring and Computer Control

Data cables must be run from both of the SP13 bulkheads mounted on the doser box to the computer that will control the robot system. Cut two lengths of CAT5 cable that are long enough to span the distance between the location where the doser box will be installed and the computer that will control the doser boxes. Strip back roughly one inch of insulation and shielding to reveal the eight wires of both cables at both ends.

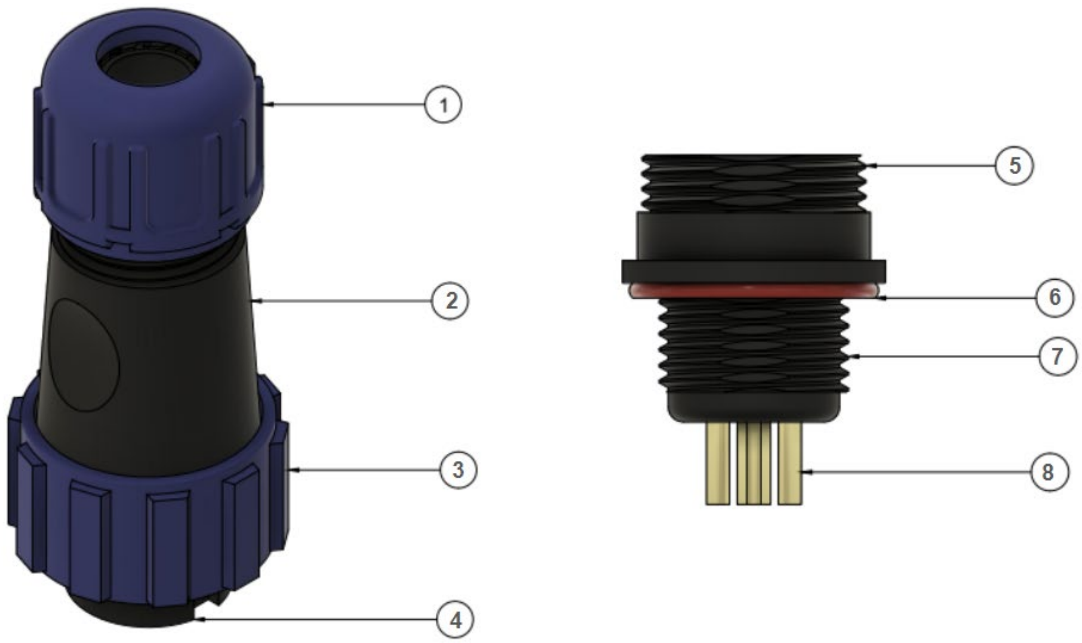

Figure 13. Image of a SP13 cable connector and bulkhead showing **(1)** cord cable gland for waterproofing, **(2)** sheath around solderable connector pins, **(3)** screw on connector, **(4)** male cable connector pins, **(5)** female bulkhead port, **(6)** bulkhead O-ring for waterproofing, **(7)** threads to attach bulkhead to device, **(8)** pins for communication wires on inside of dosing system.

To make the data cable for the Kloehn pump, one of these lengths of cable will be required as well as an SP13 cable connector (Figure 13.1-13.4), a length of DB9 cable to splice onto the other end, and a DB9 serial to USB adaptor. First, reference Table 3 to determine which wires of the CAT5 cable should be soldered to which pins of the SP13 cable connector. Unscrew the cable gland (Figure 13.1) of the cable connector and the sheath protecting the pins (Figure 13.2), revealing the pins of the connector. Solder the appropriately colored wires to the assigned pins as described in Table 3. Once complete, on the other end of the CAT5 cable, slide on the cable gland and protective sheath and screw all parts together securely to the rest of the SP13 connector to ensure the cable connector is sealed.

Table 3. *Corresponding wires of the CAT5 cable and DB9 to solder together.*

CAT5	DB9
Blue	Red
Striped Blue	Green
Brown	Black

Next, prepare the length of DB9 cable to be soldered to the other end of the CAT5 cable by stripping the terminal ½” of the three wires (Table 3). Do the same to the blue, blue striped, and brown wires of the end of the CAT5 cable as well. If available, slide a ½” segment of heat shrink to each set of wires to be soldered together. Twist the wire pairs together and solder, then apply heat shrink tubing. If heat shrink is not possible, electrical tape will work as well. On the end of the DB9 male connector, attach a female DB9 serial-USB adaptor. These USB adaptors will plug into a USB hub which is connected to the controlling computer.

The peristaltic pump wires will be connected to the controlling computer through a data acquisition chassis DAQ (cDAQ-9174) made by National Instruments, with two different data acquisition modules plugged into it. The first is a relay output module (Figure 14.6, NI-9485) for controlling the direction the peristaltic pump motors, and the second is a voltage output module (Figure 14.7, NI-9263) which controls how quickly the pump motors spin.

Every odd port on these two data acquisition modules acts as a ground port. Connect all of these ports (Figure 14.1) with small segments of black wire, including a jumper wire from one data acquisition module to the other. Screw the solid orange wire, which connects to the direction signal pin of the first pump’s motor driver, into port 0 on the NI-9485 (Figure 14.2). The solid green wire will be screwed into port 2 of the NI-9485 (Figure 14.3). The striped green wire will screw into port 0 of the NI-9263 (Figure 14.4), and the striped orange wire will be screwed into port 2 (Figure 14.5).

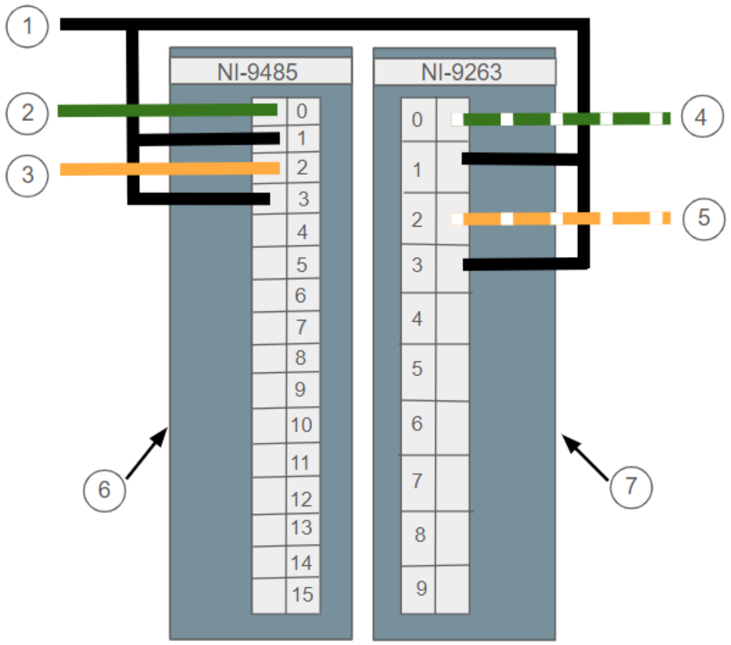

Figure 14. Wire connections for two peristaltic pumps into the cDAQ's, including **(1)** ground wires connected to every other port of both cDAQ's with a jumper wire connecting both units, **(2)** solid green wire for motor direction of first motor, **(3)** solid orange wire for direction of second motor, **(4)** striped green wire for speed of the first motor, **(5)** striped orange wire for speed of the second motor, **(6)** relay output module (NI-9485), and **(7)** voltage output module (NI-9263).

Once the dosing system is completely assembled and connected to the computer, hook up the ⅛" ID airline tubing that was mounted to the robotic arm to both of the peristaltic pumps using the ¼" to ⅛" barbs (Figure 7.2). On the other barb of the peristaltic pumps, connect each unit to the appropriate incoming water source.

3. Stir System

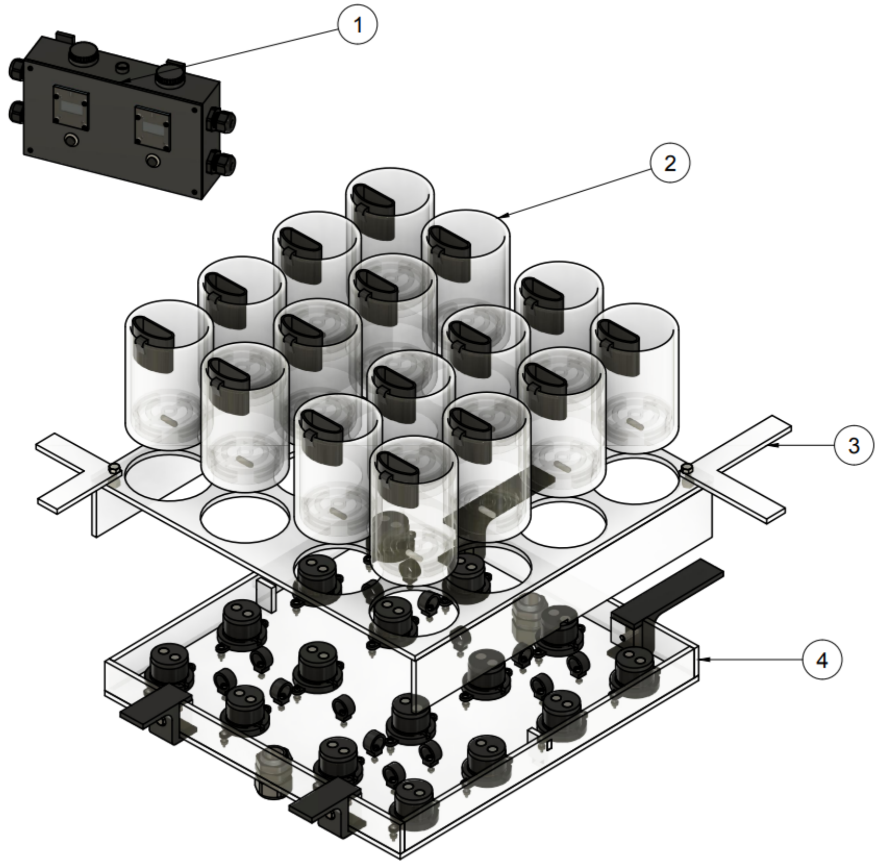

Figure 15. Stir system assembly including a **(1)** controller unit, **(2)** sixteen individual beaker assemblies, **(3)** beaker rack assembly, and **(4)** stir plate array.

This system consists of a beaker rack (Figure 15.3) with 16 identical beaker assemblies (Figure 15.2) that serve as sample vessels, a stir plate array (Figure 15.4) to be mounted below the beakers to control the magnetic stir bars inside the beakers, and a controller (Figure 15.1) to alter the speed of the motors turning the stir bars. For this section of the build, the parts from both acrylic cut sheet 1 and cut sheet 2 will be required. Additionally, the motor mounts (Figure 23.4), magnet holders (Figure 23.3), wire routing hooks, and the long and short mounting arms will need to be 3D printed. Almost all of the acrylic pieces are good to assemble right after they have been cut, with the exception of the stir array arm braces, which need to be tapped with ¼-20 threads to be compatible with the bolts that will secure the mounting arms for the array.

Beaker Assembly

Each beaker assembly consists of a glass 500 mL beaker (Figure 16.4), which has a sample stand (Figure 16.2, Figure 20), a fluid injection port (Figure 16.1), and a magnetic stir bar (Figure 16.3). There are 16 of these units per tank or stir system.

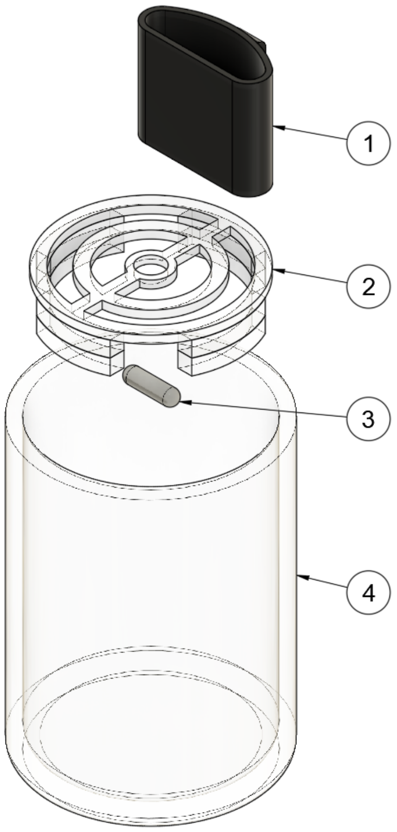

Figure 16. Beaker assembly showing the **(1)** fluid injection port, **(2)** sample stand, **(3)** stir bar, and **(4)** beaker.

The sample stand is cut out into nine individual pieces that must be glued together using acrylic adhesive: one sample stand top (Figure 17.1), and eight sample stand legs (Figure 17.2). The sample stand legs need to be doubled up and glued together to give the spinning magnetic stir bar enough clearance, giving a total of four sample stand legs after conjoining. Space the legs out evenly on the perimeter of the sample stand and glue in place as in Figure 17.

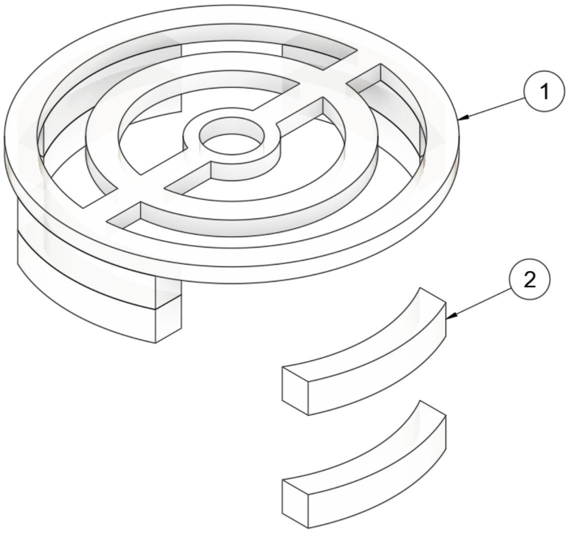

Figure 17. Sample stand showing **(1)** stand top, and **(2)** the eight stand legs cut from acrylic.

Beaker Rack

There are two different beaker rack assemblies (Figure 18, 19). The only difference between these two assemblies is the shape of the tank spacers. This is done to properly align beakers to be closer to the robot, and towards the front of the tank to avoid colliding with plumbing or other structures under the tanks. The right beaker rack (Figure 18) is designed to go to the right of the robot, and the left beaker rack (Figure 19) is designed to go to the left of the robot. These were designed specifically for the Experimental Reef Lab (displayed in Figures 1 & 2 of the manuscript). Depending on the use case of the stir system, these beaker racks may need to be modified, or may not be necessary at all

These subsystems are made mostly from laser-cut acrylic, including one beaker rack top (Figure 18.4, 19.4), two beaker rack legs (Figure 18.2, 22.2), and two tank spacers (Figure 18.1, 18.3, 19.1, 19.3) per rack set up, where the placement of the rack relative to the robot determines which spacers to use. The beaker rack top and legs are held together by acrylic adhesive, and the tank spacers are secured to the beaker rack with nylon hardware (Figure 18.5, 18.6, 19.5, 19.6).

Very little construction is required for these assemblies. The central unit of the beaker racks is always built the same way, starting by laying out the beaker rack top and two beaker rack legs on a clear and clean working surface. Connect the pieces as shown in Figures 18 and 19 and secure them using strips of painters tape along the joints. Using a syringe, apply the acrylic adhesive to the joints of the rack top and the legs. Allow to cure overnight. When the adhesive has cured, the tank spacers may be installed using the nylon hardware.

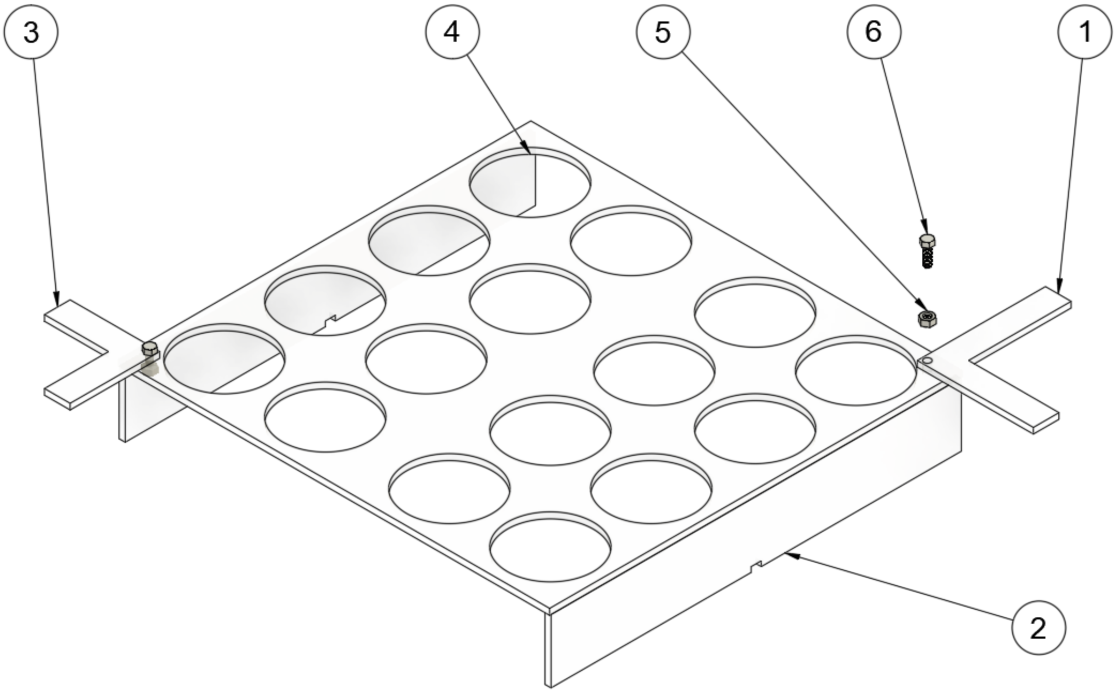

Figure 18. Right beaker rack showing **(1)** front left spacer (right tank), **(2)** beaker rack leg, **(3)** back right spacer (right tank), **(4)** beaker rack top, **(5)** nylon nut (¼-20), **(6)** nylon hex head screw (¼-20, 1 in).

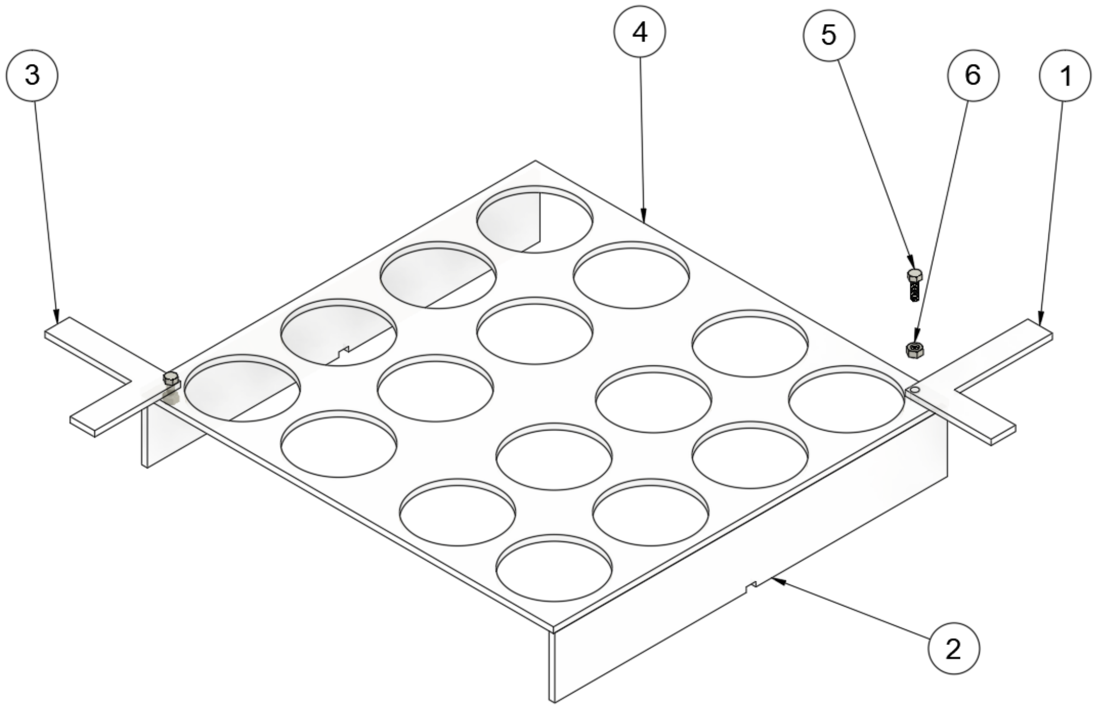

Figure 19. Left beaker rack showing **(1)** front left spacer (left tank), **(2)** beaker rack leg, **(3)** back right spacer (left tank), **(4)** beaker rack top, **(5)** nylon hex head screw (¼-20, 1 in), and **(6)** nylon nut (¼-20)

Acrylic Stir Plate Base Assembly

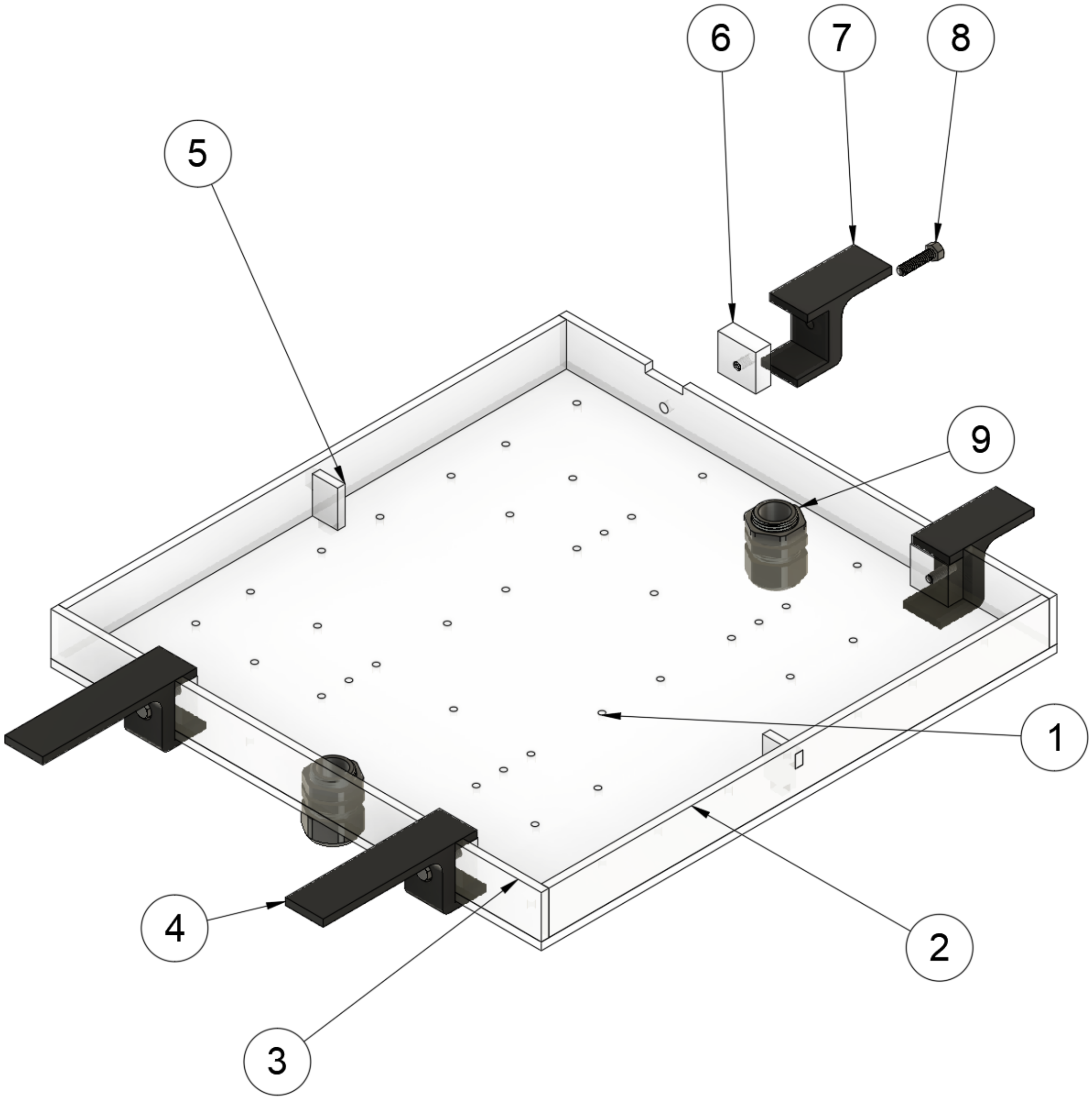

Figure 20. Stir plate housing assembly showing **(1)** stir array bottom, **(2)** stir array side, **(3)** stir array front, **(4)** short arm, **(5)** stir array brace, **(6)** arm mount (tapped, ¼-20), **(7)** long arm, **(8)** bolt (1/4-20, 1 in, stainless steel), and **(9)** cable gland (¾")

The acrylic housing for the stir plate array consists of one stir array bottom (Figure 20.1), two stir array sides (Figure 20.2), two stir array fronts (Figure 20.3), two stir array braces (Figure 20.5), and four arm mounts (Figure 20.6). These were designed specifically for the Experimental Reef Lab (displayed in Figures 1&2 of the manuscript). Depending on the use case of the stir system, these mounting arms may need to be modified, or may not be necessary at all.

To begin, attach the stir array front pieces along the edges of the bottom piece that are nearest to the large holes using strips of masking tape (Figure 21.1). Tape the stir array sides in place on the remaining two edges (Figure 21.2). Note that these front and side pieces are symmetrical and it does not matter which face points inward.

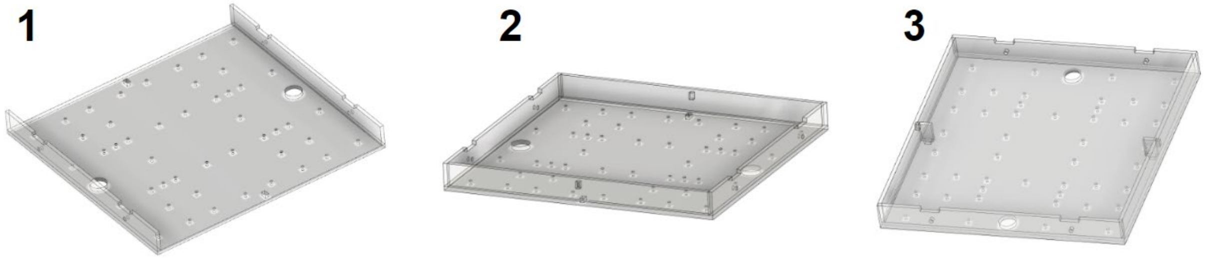

Figure 21. Assembly of the acrylic stir plate base. **(1)** Opposing sides are first glued to the bottom of the base before **(2)** the remaining two sides are added, and **(3)** vertical braces are glued in place.

When the sides and fronts are taped in position, place some extra pieces of tape along the corners where the sides and fronts meet to ensure good contact for gluing. Finally, place the braces into their slots along the sides (Figure 21.3). Using a 30 gauge syringe to draw up some acrylic adhesive from its container, apply the adhesive along the edges wherever two pieces of acrylic meet. Work one connection at a time, applying pressure for 60-90 seconds whenever a joint has been glued.

After the adhesive has finished curing, place the tapped stir array arm braces into their place at the laser-cut holes (Figure 22.1, 22.2). Screw the ¼-20 mounting bolts (Figure 20.8) into place to hold the threaded arm mount in place while it is glued. Apply the acrylic to all joints and allow them to cure.

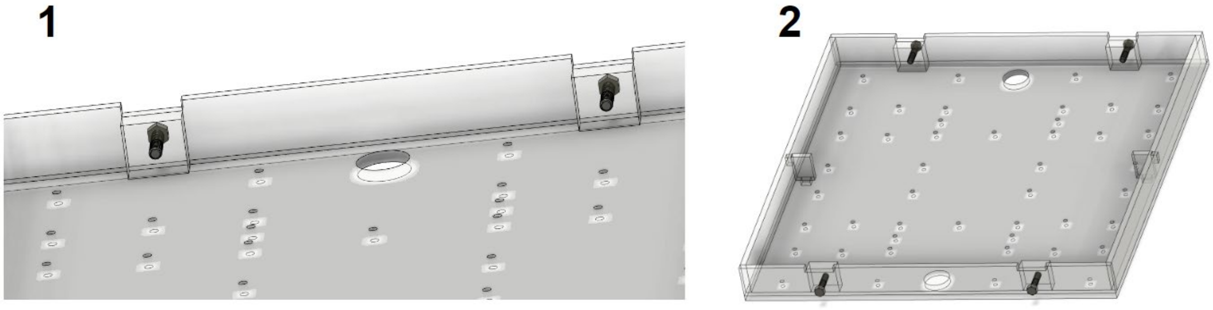

Figure 22. Assembly of the acrylic stir plate base. **(1)** Close up of arm mounts held in place with screws and **(2)** arm mounts in place while being glued.

Once the acrylic frame has cured, the cable glands (Figure 20.9) can be screwed into place in the large holes on either end of the array bottom, with the gland facing downward so it can be

loosened or tightened while the array is still installed. The mounting arms can be attached, with the shorter arms (Figure 20.7) on one side and the longer arms (Figure 20.4) on the other.

Stir Motor Assembly

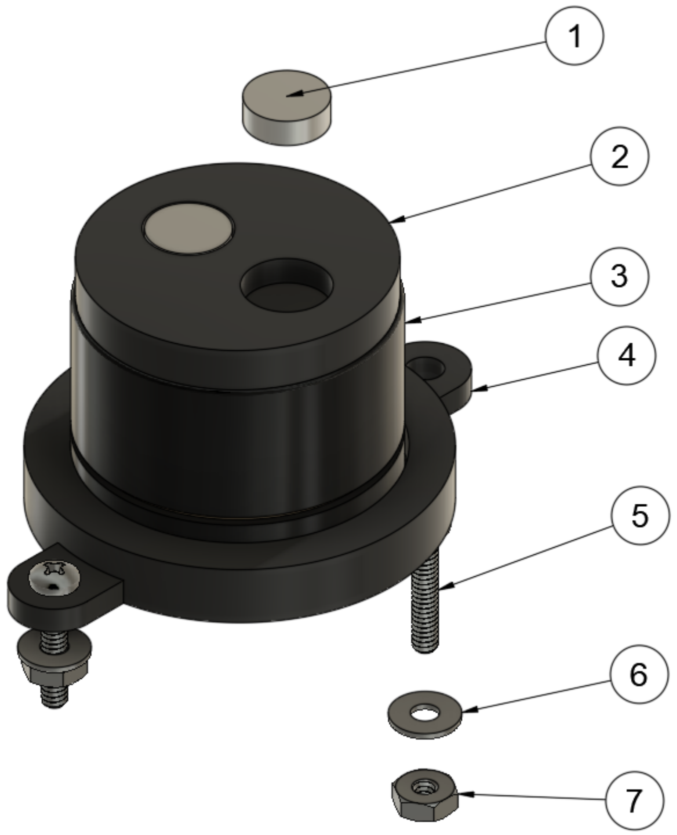

Figure 23. Stir system motor assembly showing **(1)** magnet (3/8 in diameter, 1/8 in thick), **(2)** magnet holder (3D printed, nylon), **(3)** modified fan motor, **(4)** fan motor base (3D printed, acrylic), **(5)** screw (4-40, 5/8 in, stainless steel), **(6)** washer (#4, stainless steel), and **(7)** nut (4-40, stainless steel).

The fan motors that act as the stirrers for this device (Figure 23.3, Figure 24) need to be trimmed until they are just the central cylinder of the fan. First, cut away the cage around the outside of the fan with a pair of flush cutters, being careful not to clip the wires for the fan (Figure 24.1). Once the outside cage is off, there will likely be some small nubs of plastic coming from where the cage supports were clipped off. These will need to be trimmed back for the fan motor to fit into its 3D printed motor mount (Figure 24.2). Once the base has been trimmed, cut the fan blades off as well (Figure 24.3, 24.4). Repeat this process for the remaining fan motors until there are 16 fully trimmed motors.

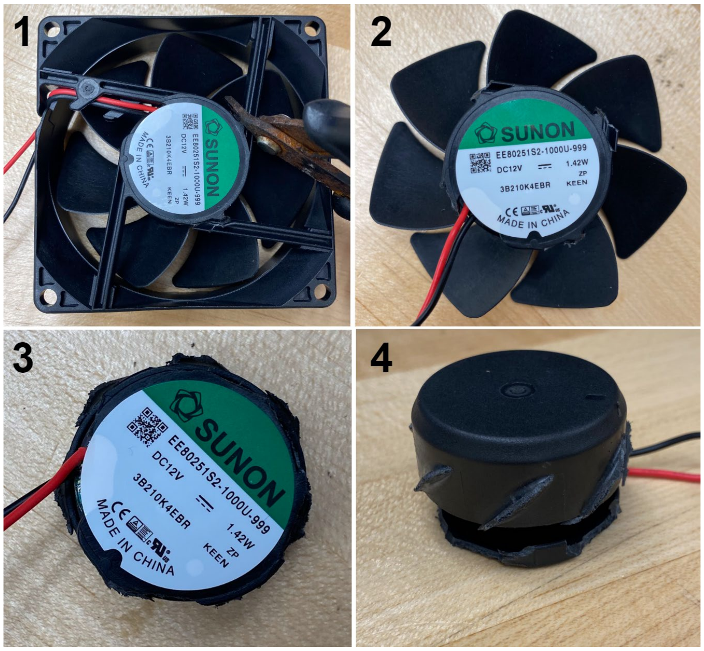

Figure 24. Fan motor modification. **(1)** Fan motor before trimming, **(2)** fan motor with outside cage removed, **(3)** fan motor with blades removed, and **(4)** fully trimmed fan motor for stir plate.

The wires for the fan motors need to be extended to be able to reach the stir plate controller. For every motor, cut two wires of 3 feet in length. Solder the 3-foot lengths of wire to the positive and ground wires of the motor. If a power source is available, testing the motors for functionality is advised by applying 12V DC to the wires. Apply heat shrink or electrical tape to the connection point of the wires where they were soldered together.

Put some scrap cardboard or other protective material on a flat work surface, and line up the 16 motor mounts (Figure 23.4). Mix two-part epoxy in a small disposable container, and apply a dot of epoxy approximately 5 mm in diameter to the center of the motor mounts. Take the trimmed fan motors (Figure 23.3) and fit them inside the center of the motor mounts on top of the epoxy. The wires should nestle into the groove on the motor mount to prevent strain on the wires' connection points. Allow the epoxy to cure overnight. While the motors are curing to their mounts, add another small 5mm dot of epoxy to the top. Place the magnet holder (Figure 23.2) on top of the fan motor as close to the center as possible. Spinning the motor helps to visualize if the magnet holder is properly centered. Tape this in place to ensure the magnet holder does not move while curing.

Allow 8-12 hours for the epoxy to cure, sufficiently affixing the motors to their mounts and the magnet holders to the tops of the motors. Following that, apply a small 2-3 mm dot of epoxy in the indentations of the magnet holders. Place the neodymium magnets (Figure 23.1) into the epoxied indentations of the magnet holders, making sure that opposite poles are facing upward for each indentation. Opposite poles must face upward for the magnets to interact correctly with the stir bar. Once the magnets are in place, apply a piece of tape over them while the epoxy

cures. These magnets are very strong, and over time they will pull towards each other and may even pull themselves free of the indentations if they are not secured. Allow the epoxy to cure overnight. Once the individual stirrer units have been fully epoxied together, they can be fastened to the acrylic housing using the 4-40 screws (Figure 23.5), nuts (Figure 23.7), and washers (Figure 23.6). For reference, Figure 23 shows the layout the stir motor assemblies should be fastened in.

Cable Router Assembly

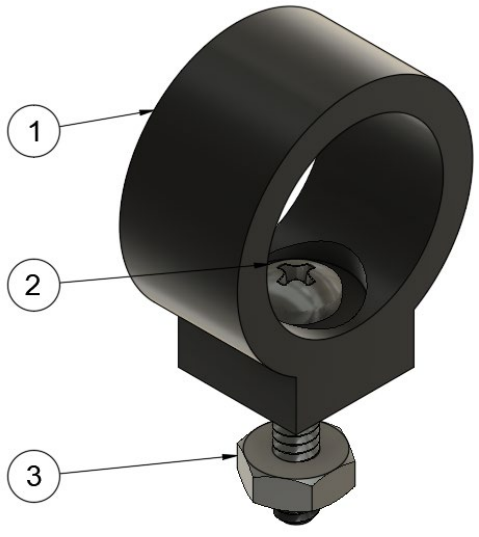

Figure 25. Cable router assembly showing **(1)** cable hook (3D printed, nylon), **(2)** screw (4-40, 5/8 in, stainless steel), and **(3)** nut (4-40, stainless steel).

A series of cable routers are installed on the stir plate array to organize the cables from the stir motor units. These cable routers consist of a 3D printed cable hook (Figure 25.1), and 4-40 screws (Figure 25.2) and nuts (Figure 25.3) to secure the cable hooks to the bottom of the stir plate array. Reference Figure 26 for cable router placement and use.

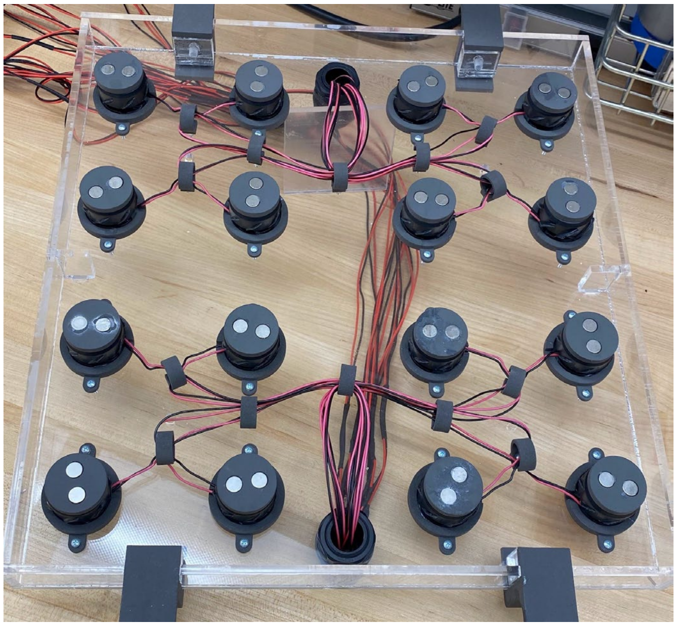

Figure 26. Photo of stir array showing wire routing paths in use.

Stir Plate Controller

Two circuit board units (Figure 27) are required to build one stir plate controller (Figure 28). Each circuit board unit has a custom circuit board (Figure 27.6), an LCD screen for menu display (Figure 28.7), an IR sensor to communicate with the controller (Figure 28.8), and a pushbutton to interrupt the stir plate program while it is running (Figure 28.1). The 3D printed parts required for this part of the build are a controller box (Figure 28.9), a controller lid (Figure 28.2), two controller port screws (28.10), and two controller tank mounts (Figure 28.17). The two controller windows should be cut out of quarter inch acrylic and are included on cut sheet 1.

Circuit Board Assembly

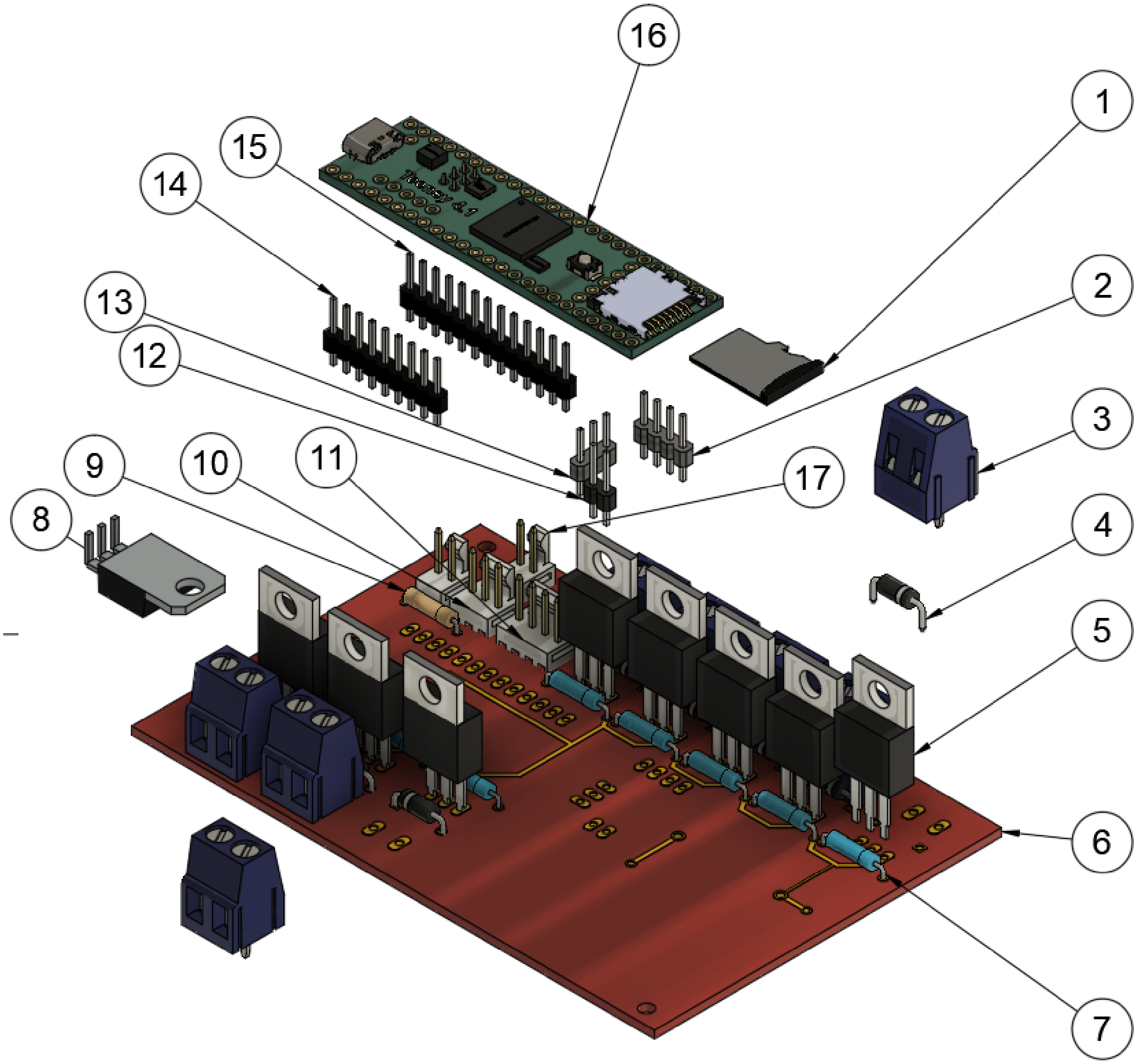

Figure 27. Stir plate controller circuit board showing **(1)** micro SD card, **(2)** header (4 pins), **(3)** screw terminal (0.2 in pitch, 2 position), **(4)** diode (1N4004), **(5)** mosfet (N-channel, IRLB8721PBF), **(6)** custom stir controller circuit board, **(7)** resistor (10k Ω), **(8)** voltage regulator (5 v, 1.2 A), **(9)** resistor (3.3k Ω), **(10)** Molex KK connector (3 pin male), **(11)** Molex KK connector (4 pin male), **(12)** header (2 pin), **(13)** header (3 pin), **(14)** header (9 pin), **(15)** header (13 pin), **(16)** Teensy 3.5 microcontroller, **(17)** Molex KK connector (2 pin male).

To start, solder the headers (Figure 27.2, 27.12 - 27.15) for the Teensy (Figure 27.16) onto the circuit board (Figure 27.6). It is advantageous to rest the Teensy (Figure 27.16) on the top side of the headers while they are being soldered into place from the bottom. This helps to ensure

the headers remain perpendicular to the board and the Teensy will fit onto the headers correctly.

Solder on all resistors and diodes (Figure 27.4, 27.7, 27.9). Resistors are not directionally sensitive, however diodes need to be placed in the correct orientation to regulate current correctly. The line on the diode body should go to the same end as the line on the board silkscreen if it was purchased online, but if the circuit board was milled by the builder, follow the orientation of the diodes in Figure 27.

The male Molex KK connectors should be soldered on next (Figure 27.10, 27.11, 27.17), along with the screw terminals (Figure 27.3).

The N-channel mosfets (Figure 27.5) and voltage regulator (Figure 27.8) are soldered on next. The metallic back of the mosfet should be positioned in line with the thick white line of the silkscreen on the board, following the reference in Figure 27. The voltage regulator should be installed on the bottom side of the board, with the pins bent at a 90 degree angle so the back of the voltage regulator rests along the circuit board.

The Teensy microcontroller (Figure 27.16) should be installed last. Place the Teensy on the headers that were soldered in at the beginning of board construction and solder along the top of all the header pins, being careful not to damage components on the Teensy board.

Controller Box Construction

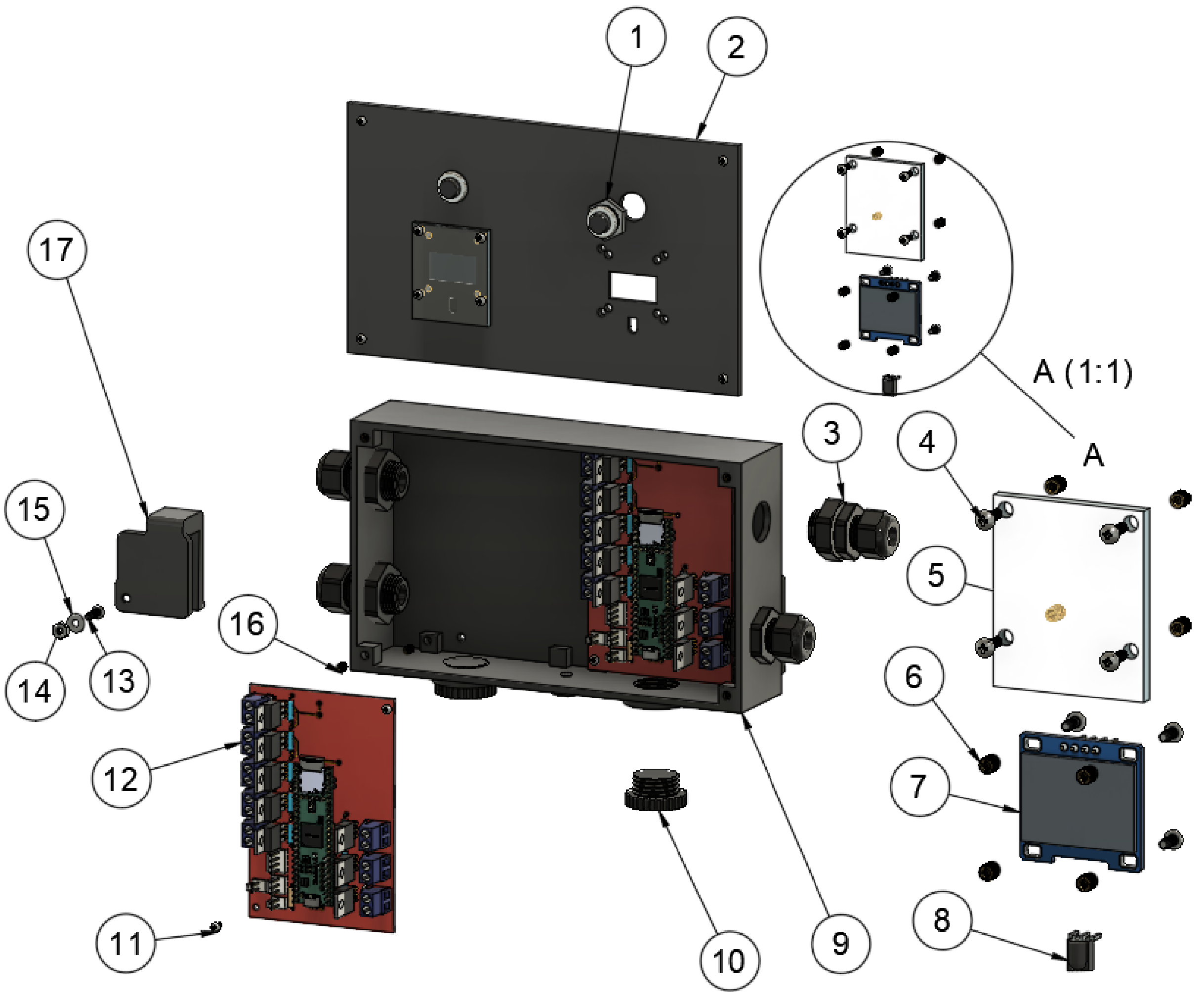

Figure 28. Stir system controller assembly showing **(1)** momentary push button, **(2)** stir controller lid (3D printed, nylon), **(3)** cable gland (3/8 in), **(4)** screw (2-56, 3/16 in, stainless steel), **(5)** acrylic OLED window, **(6)** threaded insert (2-56, brass), **(7)** OLED screen, **(8)** IR sensor, **(9)** stir controller box (3D printed, nylon), **(10)** stir controller screw (3D printed, nylon), **(11)** screw (2-56, 3/16 in, stainless steel), **(12)** assembled stir controller circuit board, **(13)** screw (4-40, 3/8“, stainless steel), **(14)** nut (4-40, stainless steel), **(15)** washer (0.125” ID, 0.312” OD, stainless steel), **(16)** threaded insert (2-56, brass), and **(17)** stir controller tank clip

The single power source for the circuit boards is routed through the bottom of the controller box, as seen in Figure 29. It needs to be split into two different power cords, one for each board. Do so by soldering an additional length of wire to both the red and black wires of the waterproof barrel jack. Crimp the ends of all four wires with Molex KK crimps. Slip the waterproof barrel jack into the appropriate place on the controller housing, so the wires that were soldered and crimped are on the interior of the box, and the barrel jack is on the exterior

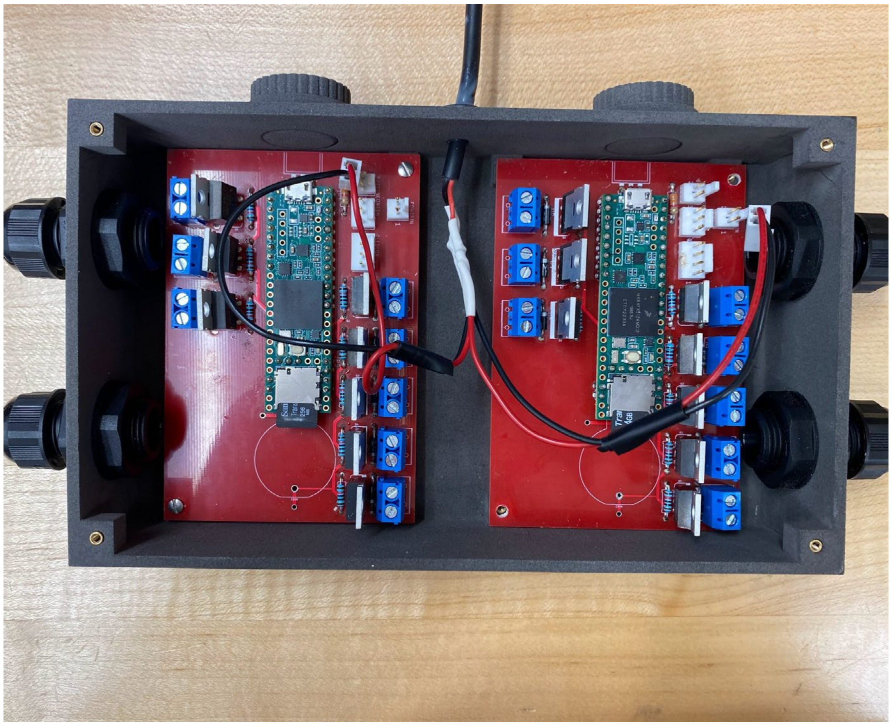

Figure 29. Photo showing potted waterproof power cable routed to inside of the controller box where it diverts to two separate power cables to supply voltage to both circuit boards

Turn the controller box upside down. There should be a “cup” where the barrel jack enters the controller housing. Mix some two part epoxy and deposit in this “cup”. When the epoxy cures, this will keep the barrel jack in place and seal this part of the box.

After the epoxy has cured, the threaded inserts for the controller box and controller lid should be pushed into place, as shown in Figure 30. Using a hard flat object such as a scrap piece of acrylic to push these in is recommended to avoid scratching the controller box or the threaded inserts themselves.

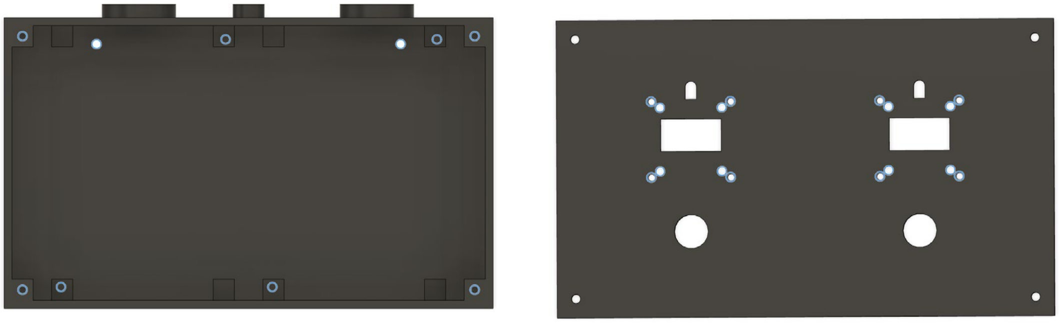

Figure 30. Controller box where highlighted areas represent mounting holes for 2-56 threaded inserts

Finally, the IR sensor, pushbutton, and OLED screen should be affixed to the controller lid. Solder the headers onto the OLED, and then bend the headers at a roughly 30 degree angle to the board. Solder wires to the pins of the pushbutton. The OLED can be screwed into place using the appropriate threaded inserts. The pushbutton can be screwed into place and affixed with the nut the button comes packaged with. The IR sensor will need to be epoxied into place. Position the lid on a flat surface. Bend the pins of the IR receiver at a 90 degree angle as close to the body of the IR receiver as possible, as seen in Figure 31. Place the receiver in the appropriate position on the lid and epoxy it into place. Let it cure overnight.

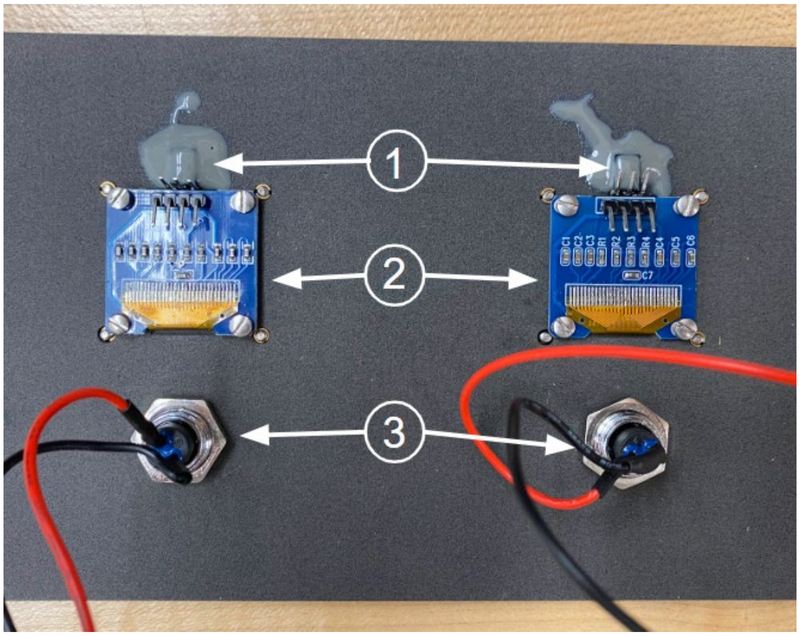

Figure 31. Photo showing the **(1)** IR sensors epoxied in place, **(2)** OLED screens screwed into place, and **(3)** push buttons installed

For the wiring to the IR receiver and OLED, make cables with Dupont-style connectors at one end and Molex KK style connectors at the other. See Figure 32.1 and 32.2 for examples of the OLED and IR cables, respectively.

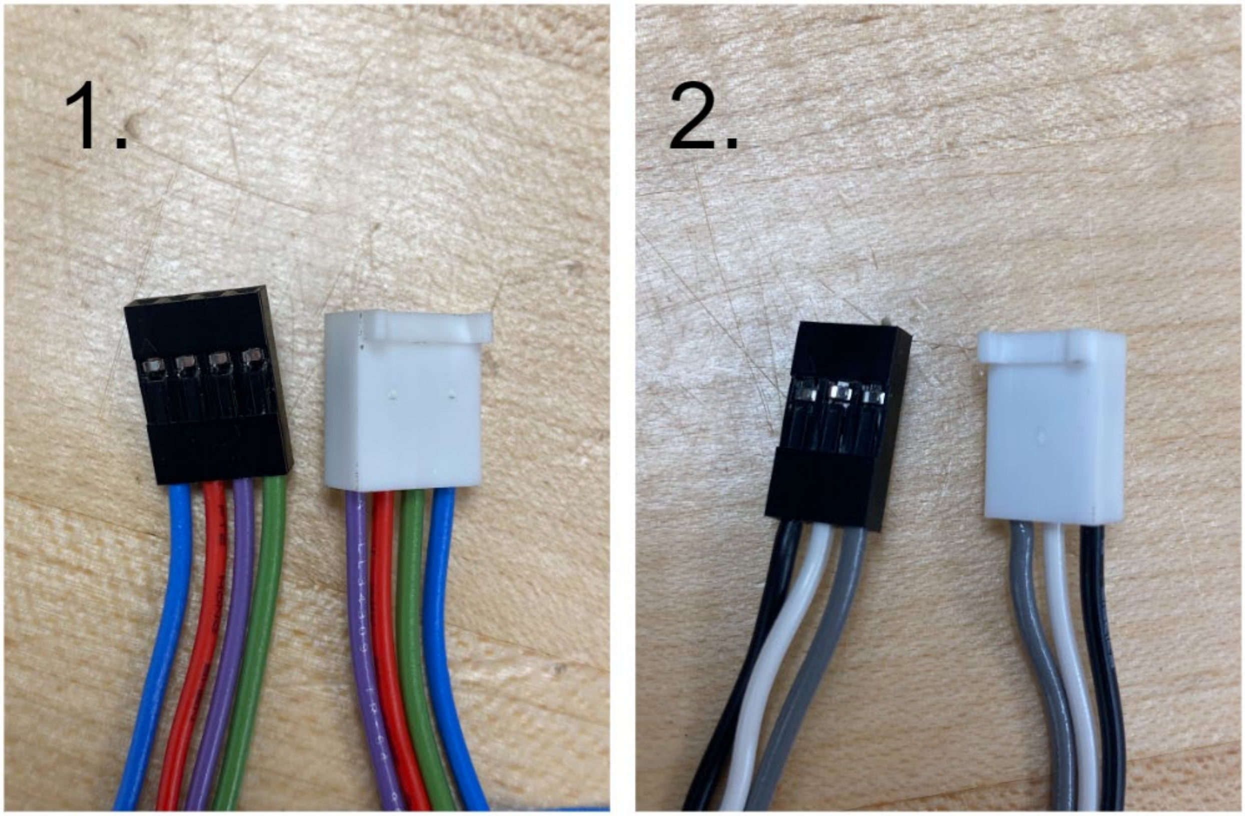

Figure 32. Images of the wires and connectors for the **(1)** OLED screen and **(2)** IR sensor

Software Download and Setup

Follow the step-by-step instructions on the Teensy website to download and install the Arduino and Teensyduino software onto a computer. Ensure that the version of Arduino that is installed is compatible with the Teensy software, the Teensyduino install wizard states compatible Arduino versions on the first page of the install setup.

Once the software is installed, download the stir plate code from the STAR system source file repository (https://zenodo.org/doi/10.5281/zenodo.10048123). Move the code into a folder titled the same as the code file. For example, if the code file is called “starStirCode.ino”, it should be inside a folder called “starStirCode”. Move this folder to the documents tab > “Arduino” > “Arduino libraries”.

Arduino Libraries

This code requires the use of several libraries to run properly, which are listed in Table 4.

Table 4. *Arduino Libraries for Stir System Controller needed for the code, with their purposes and authors.*

Library	Purpose	Author
Wire.h	Data communication	A
Adafruit_GFX.h	Adafruit OLED display	Fried, L; Adafruit
Adafruit_SSD1306.h	Adafruit OLED display	Fried, L; Adafruit
IRremote.h	IR communication	Kahn,R; Shirriff, K; et al.
SdFat.h	Read and write to SD card	Greiman, B.
SPI.h	Serial communication	Maglie, C; Stoffregen, P; Kooijman, M

To include these libraries, click the sketch tab, go to “Include Libraries,” and select “Manage Libraries”. When the library manager pops up, search for each library and download the most recent version of each. This will download the library to the “starStirCode” folder the code was placed inside.

The Adafruit_SSD1306 library is responsible for the output to the OLED screen, and the resolution for the OLED being used needs to be modified inside the code of the library. To do this, find the folder for the library. On a PC, the file path should be Arduino > hardware > teensy > avr > libraries > Adafruit_SSD1306. On Mac, right click the Teensyduino icon and select “Show Package Contents” > Contents > Java > hardware, at which point the directions should be the same. Open the file titled “Adafruit_SSD1306.h” with the text editing program of the builder’s choice. Scroll down to the following section:

```
//ONE of the following three lines must be #defined:
//#define SSD1306_128_64 ///< DEPRECATED: old way to specify 128x64 screen
#define SSD1306_128_64 ///< DEPRECATED: old way to specify 128x32 screen
//#define SSD1306_96_16 ///<DEPRECATED: old way to specify 96x16 screen
```

The default resolution of this library is for a screen resolution of 128 x 32, but the screen resolution used in this code is for 128 x 64. To remedy this, the comments should be removed from the 128_64 line, and new comments should be added before the 128_32 line, like so:

```
//ONE of the following three lines must be #defined:
#define SSD1306_128_64 ///< DEPRECATED: old way to specify 128x64 screen
//#define SSD1306_128_64 ///< DEPRECATED: old way to specify 128x32 screen
//#define SSD1306_96_16 ///<DEPRECATED: old way to specify 96x16 screen
```

Once this is taken care of and saved, the libraries are good to go, and the code may be uploaded to the Teensy.

Code Upload

To begin, open the Teensyduino software. Under Tools > Board, choose Teensy 3.5, this step should only be necessary the first time the program is opened. From the File tab, open the starStirCode file that was saved in the library folder that was created in part II. Once the code has been opened, click the verify button in the upper right corner of the screen to compile the code.

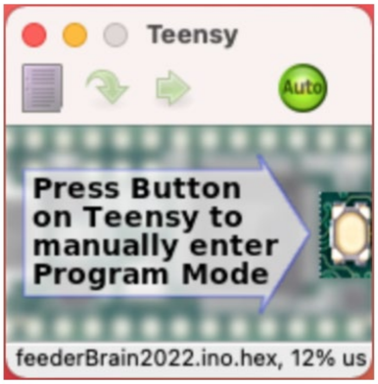

Figure 33. Image of the Teensy code upload window

Once the code has been successfully compiled, a new window that looks like Figure 33 should pop up. This window is how the code is sent to the Teensy. Connect the computer being used to the Teensy via a USB to micro USB cable. Once connected, press the beige button next to the SD card slot. This should trigger the Teensyduino program to send the compiled code to the Teensy. The OLED screen should power on at this point and say “MicroSD Card Not Detected”.

Micro SD Installation

The controller is designed to retain individual power settings in case of power interruption. Upon changing the voltage settings on the controller and selecting “ENTER SET”, a micro SD card inserted into the Teensy will save and retain the settings the user has selected. In the case of a temporary power interruption, once the controller is reconnected to power it will automatically

read the user input settings from the micro SD and start up with them. In order for the controller to work as intended, a micro SD card that has been formatted correctly is necessary.

Using a text editor (e.g TextEdit on Mac, Notepad on PC, Nano on Linux), create a file that has eight 0's separated by commas on a single line (Figure 34). Save this file as .TXT file, with the file name "sampleParam.TXT". Once saved, move this file onto the Micro SD for the controller. When this SD card is slotted into the Teensy and power is applied to the controller, the text saying "MicroSD Card Not Detected" should no longer be displayed.

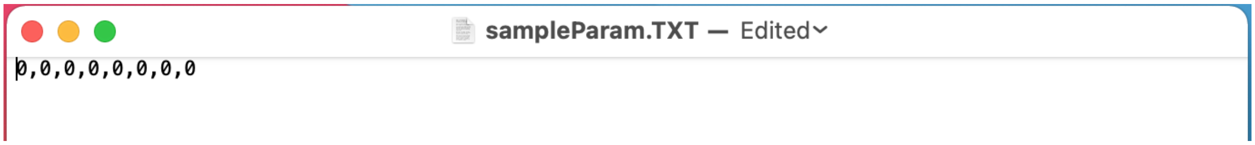

Figure 34. Image showing details of the file to be saved to the micro SD card

Software Usage

The controller is programmed to start applying voltage when power is connected to the device. This is so the motors can start back up without user intervention in the case of power interruption. To wake the controller, the button must be pushed to get the main menu screen to pop up. Here, there should be an array of 8 numbers, 0's if this is the first time the software is being used. Use the IR remote to navigate to each number, and use the up or down buttons on the remote to raise or lower the voltage of that particular motor, where higher voltage increases spinning speed and lower voltage decreases speed. Once the settings are at the desired voltages, navigate to the bottom of the screen and select "Enter Set". This should write the settings to the sampleParam file on the SD card, the OLED screen should go blank, and the motors should start spinning at the user-programmed speeds.

4. Cellular Watchdog

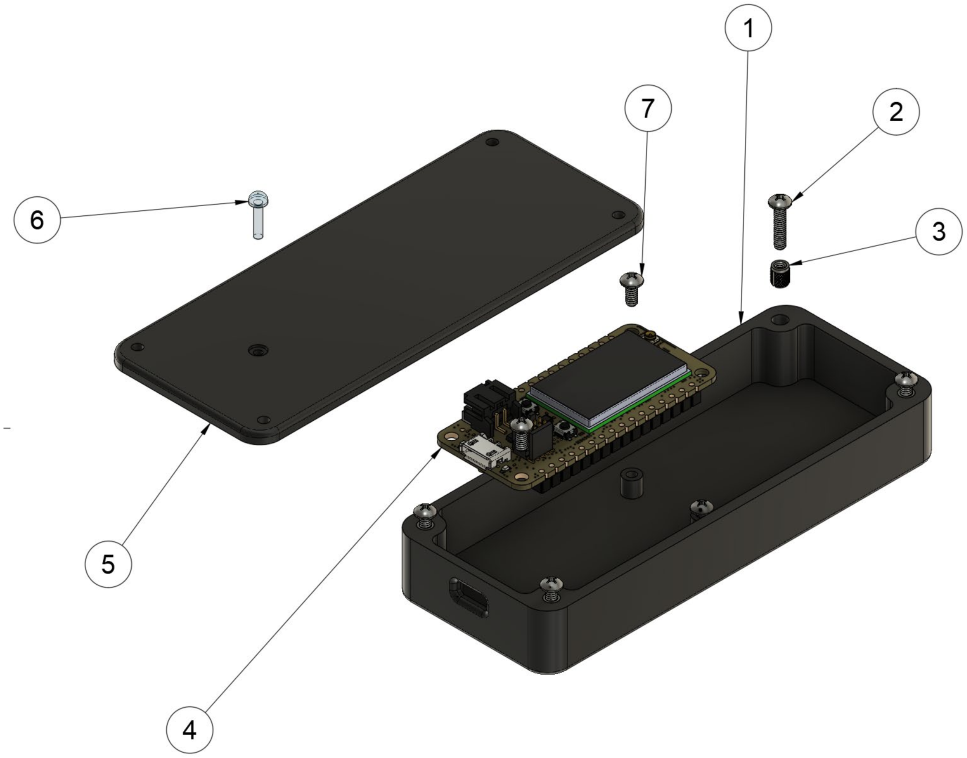

Figure 35. Cellular watchdog including **(1)** 3D printed housing base, **(2)** screw (4ct 2-56, 3/8” long, stainless steel), **(3)** threaded insert for plastic (4ct, 2-56, 0.156” long, brass), **(4)** Particle Boron device, **(5)** 3D printed housing lid, **(6)** 3D printed translucent light cover, **(7)** screw (4ct 2-56, 3/16” long, stainless steel).

The cellular watchdog is composed of a Particle Boron device (Figure 35.4) affixed inside a 3D printed housing (Figure 35.1, 35.5). The cellular watchdog is not required for the operation of the STAR system, but can be useful for monitoring activity and performance diagnostics of the system. Press the threaded inserts (Figure 35.3) into place on the corners of the housing base (Figure 35.1). Screw the Boron to the housing base with 2-56 screws (Figure 35.7). Attach the antenna (Figure 36.1) to the appropriate place on the Boron (Figure 36.2) and tuck it neatly inside the housing base so as not to interfere with the housing lid (Figure 35.5) when it is clipped into place and fastened with 2-56 screws (Figure 35.2). Plug the device into the controller computer using a USB to Micro USB cable through the port on the housing base. Follow the instructions included with the Particle Boron to prepare it for operation.

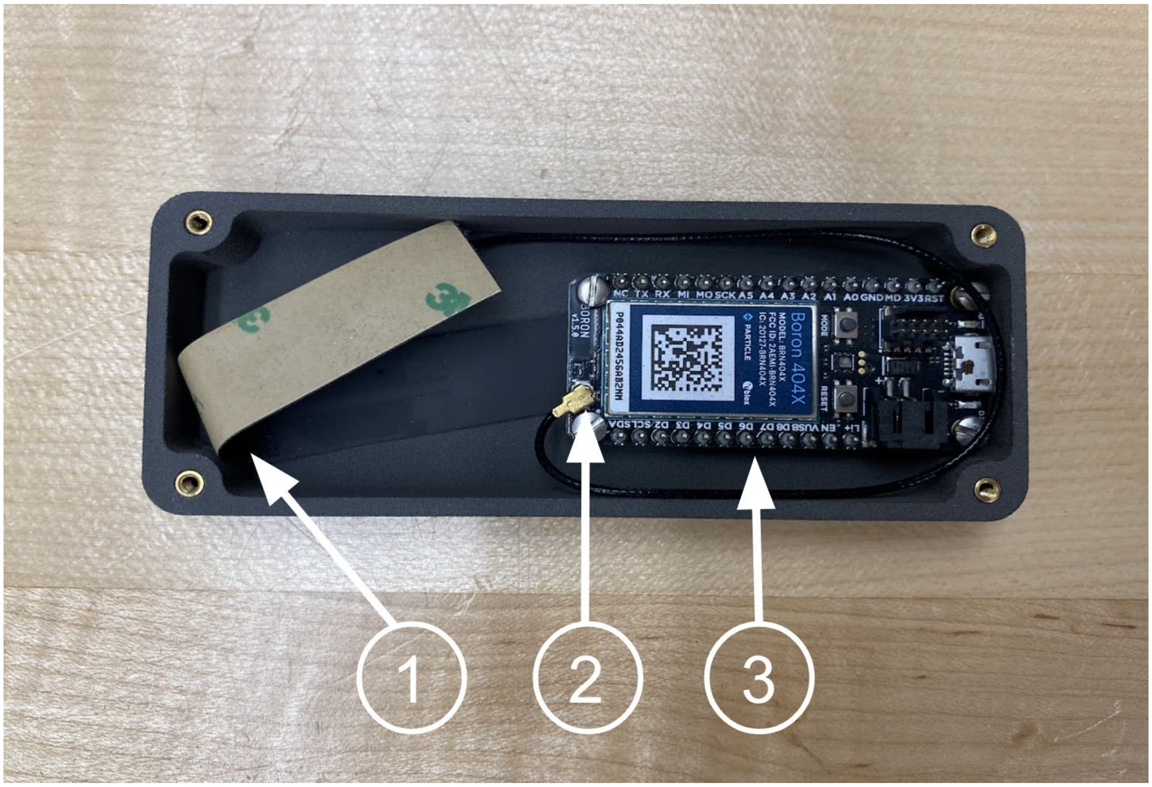

Figure 36. Image of an **(1)** antenna **(2)** connected to the **(3)** Particle Boron device.

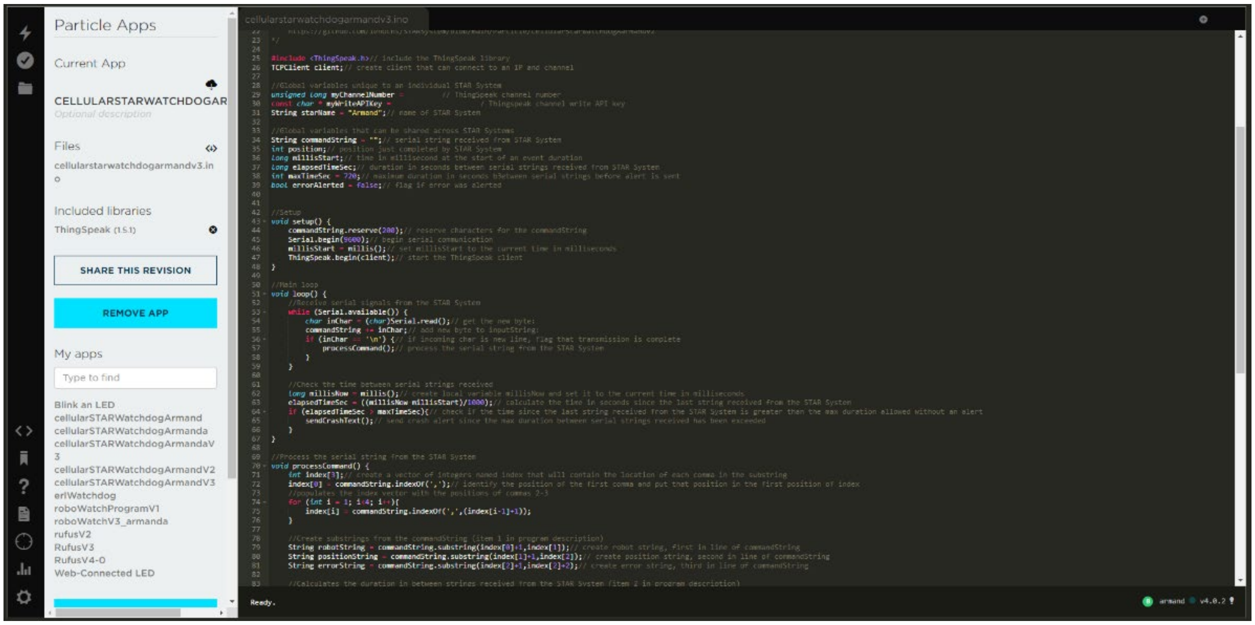

Figure 37. Image of Particle's Web IDE (https://build.particle.io/build/).

Download the code for the watchdog from the source file repository. Log in to Particle's Web IDE (Figure 37, https://build.particle.io/build/). Register devices in the Devices tab (Figure 38) in

the bottom left menu. Here all devices that are registered are displayed by name (Figure 38.4). Devices that are starred (Figure 38.5) are currently selected in the IDE, and devices that have light blue circles to the right of their name (Figure 38.6) are currently connected and online.

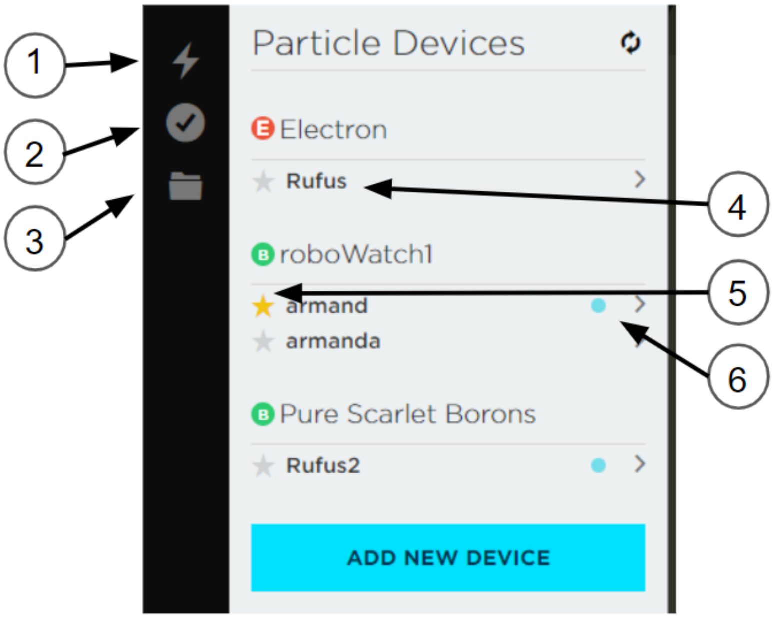

Figure 38. Image showing the main menu as well as the Device tab of the Particle Web IDE, including **(1)** button to flash code to device, **(2)** button to verify code, **(3)** button to save, **(4)** registered device name, **(5)** star indicating the active device selected, **(6)** indicator for devices that are connected and online

To upload the code to the Boron from the IDE, first make sure that the board selected for changes is starred in the Devices tab, and is highlighted in green in the bottom right corner of the IDE screen. With the desired code loaded on the IDE, select Verify (Figure 38.1) in the upper left hand menu. If the code is accepted by the program, a banner notification at the bottom of the screen will appear that says the code has been verified. The device can now have the code flashed to it by selecting the Flash icon (Figure 38.1), and it should start functioning right away.

5. Computer Setup and Wiring

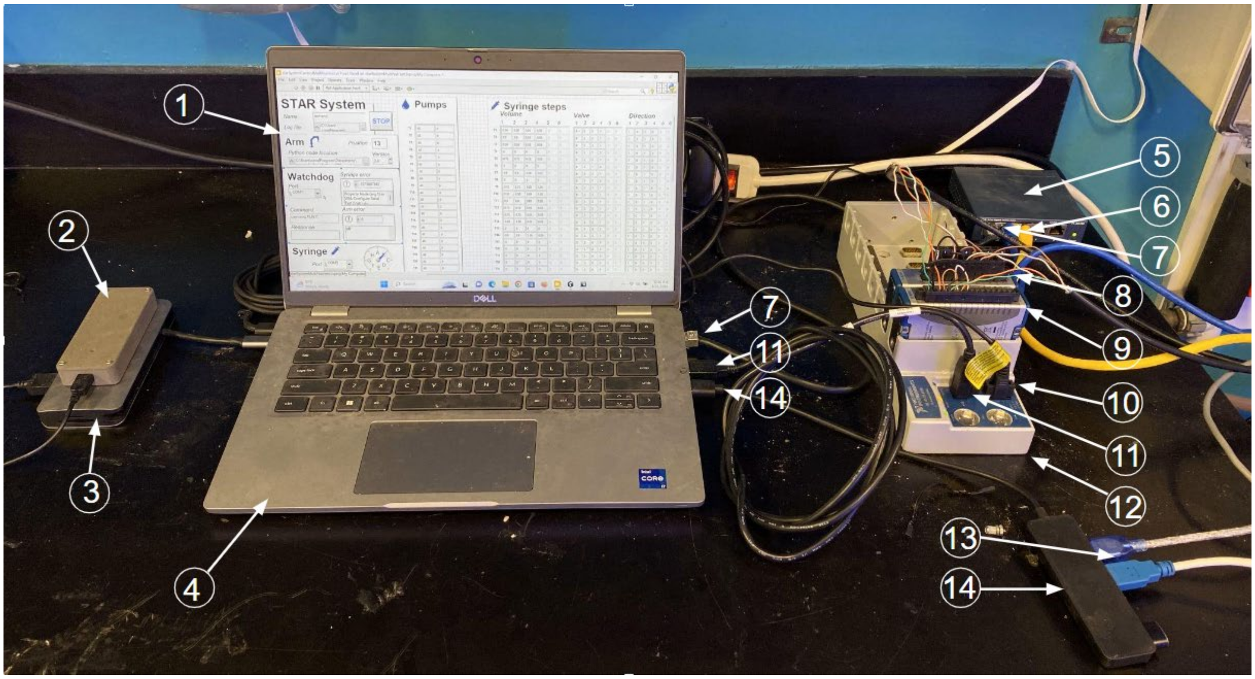

Figure 39. Photo of system control station including **(1)** Labview control GUI, **(2)** cellular watchdog, **(3)** multi-port USB hub for connecting multiple watchdogs, **(4)** system control laptop, **(5)** ethernet switch for controlling multiple devices via ethernet, **(6)** ethernet cables coming from two robots, **(7)** ethernet cable connecting the switch to the system control laptop, **(8)** voltage output module (NI-9263), **(9)** relay output module (NI-9485), **(10)** cDAQ chassis power cord, **(11)** cDAQ chassis USB communication cable, **(12)** cDAQ chassis, **(13)** serial DB9 to USB adaptors leading to two Kloehn pumps, **(14)** multi-port USB hub for connecting multiple USB devices.

The controlling computer (Figure 39.4) for the STAR system will need several USB ports or, more likely, one or two USB hubs (Figure 39.3, 39.14) to allow the computer to connect to the other subsystems including the cellular watchdog (39.2) which is connected with a USB to micro USB cable, as well as the serial to USB adaptors (Figure 39.13) for the syringe pump of the dosing system. The peristaltic pumps of the dosing system are wired into two cDAQ modules (Figure 39.8, 39.9), which in turn are plugged into a cDAQ chassis (Figure 39.12). The chassis will connect to the controller computer (Figure 39.11) with a USB to USB-A cable and will also need to be plugged into a power source (Figure 39.10). The ethernet cord of the robot (Figure 39.6) will either plug directly into the controller computer if only using one robot, or in the case where there are more robots than ethernet ports an ethernet switch (Figure 39.5) will be needed with a separate ethernet cord (Figure 39.7) to connect the switch to the computer.

The STAR control GUI (Figure 39.1) is broken down into a few sections. To learn more about the operation of the GUI, reference the Operation instructions section of the main manuscript.

6. Robot Setup and Calibration

The UFACTORY xArm robot will require some setup steps before it is ready to be used with the STAR System LabVIEW code. Each xArm should arrive with a quick start guide that goes over the basic steps of making the computer compatible with the robot's software, UFACTORY Studio, which is necessary for the initial setup of the robot and can be downloaded from UFACTORY's website (https://www.ufactory.cc/ufactory-studio/).

Connect the robot to the control box and computer as instructed in the quick start guide, or for more detailed instructions on installation of the xArm, visit UFACTORY's download center and download the user manual for the xArm Robot (https://www.ufactory.cc/download/). Plug the power cable for the control box in. Connect the robotic arm to the linear motor using the two cables that are run along the cable track of the linear motor. There is a large power supply cable and a smaller signal cable. The linear motor will connect to the control box by three different cables. One large cable will attach at the end of the linear motor and lead to the front of the control box, and the two cables that connect to the robotic arm and are routed through the cable track will be plugged into the back of the control box. Finally, an ethernet cable will connect the control box to the computer.

IP Address Configuration

Once all of the robot system's hardware is properly connected, plug the control box in and flip the power switch. This should result in some initial beeping coming from the control box. If nothing happens, ensure the emergency stop button on the top of the control box is not activated. Once the control box is plugged in and turned on, wait until there is a second round of beeping, which indicates the robot is ready to connect.

In order for the computer to work with the robot system, the IP address of the computer needs to be configured to match the LAN environment of the control box. For a PC running a Windows operating system, this is done by navigating to the Network and Sharing Center (Figure 40). Under "View your active networks" click on the ethernet connection option that corresponds to the robotic arm.

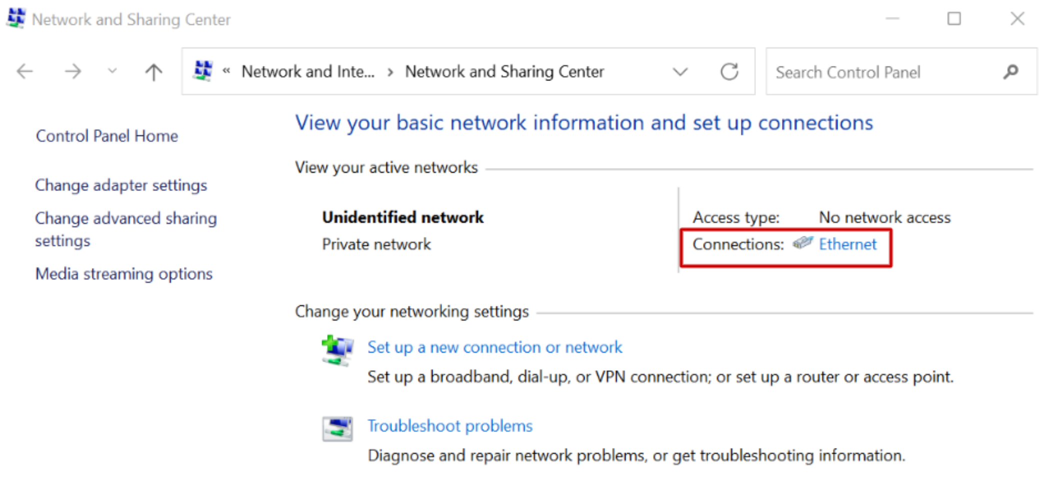

Figure 40. Image of the Network and Sharing Center tab with the ethernet connection for the robot highlighted in red.

A window titled “Ethernet Status" will pop up (Figure 41). Click the properties tab in the bottom left corner.

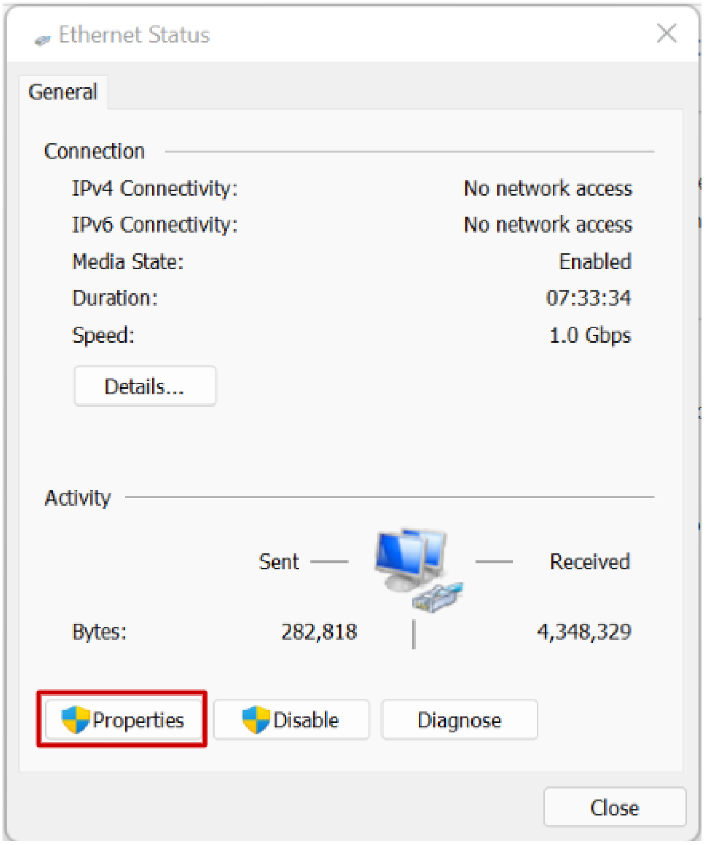

Figure 41. Image of the Ethernet Status pop-up with the Properties tab highlighted in red.

Another window will pop up, titled “Ethernet Properties” (Figure 42). Select “Internet Protocol Version 4 (TCP/IPv4)” and then click on the Properties button again.

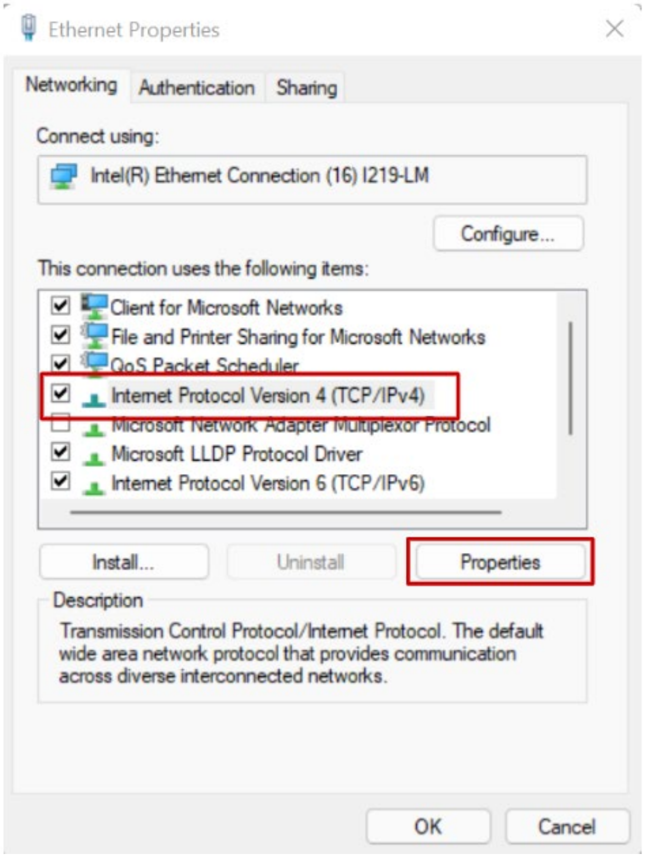

Figure 42. Image of the Ethernet Properties pop-up with the IPv4 connection and the respective properties tab highlighted in red.

In the window that appears (Figure 43), select “Use the following IP address.” Fill in the IP address so that the first three sections of numbers match the IP address of the control box, but the last section of numbers are different from the control box (i.e. if the control box IP address is 192.168.1.123, an appropriate IP address for the computer would be 192.168.1.234). The IP address of the control box can be found printed on the back of the control box itself. Fill in the rest of the information as depicted in Figure 43. Select OK to save changes.

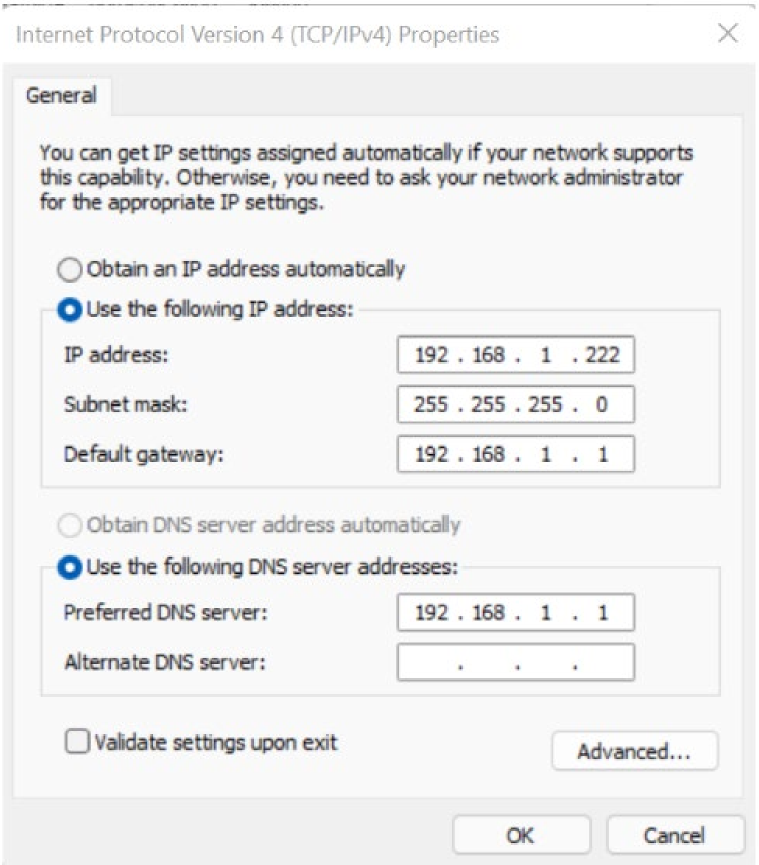

Figure 43. Image of the IP Properties tab with an example IP address.

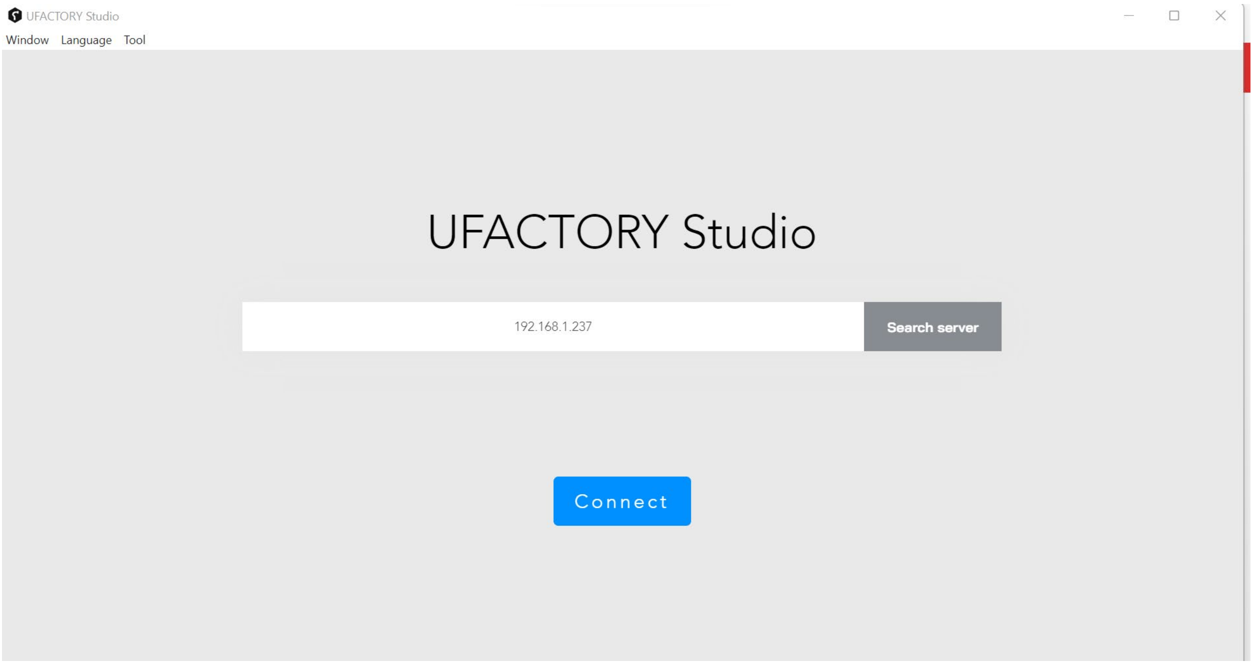

Figure 44. Image of the opening page of the UFACTORY Studio software.

Once the IP address of the computer has been set, open the UFACTORY Studio software (Figure 44). Upon opening the program, the user will be prompted to enter the IP address of the controller box and select “Connect”. Note that if the user has not turned on the control box and heard the second round of beeping, the program will fail to connect to the robot. If the connection is successful, the user will be directed to the home screen of the program (Figure 45).

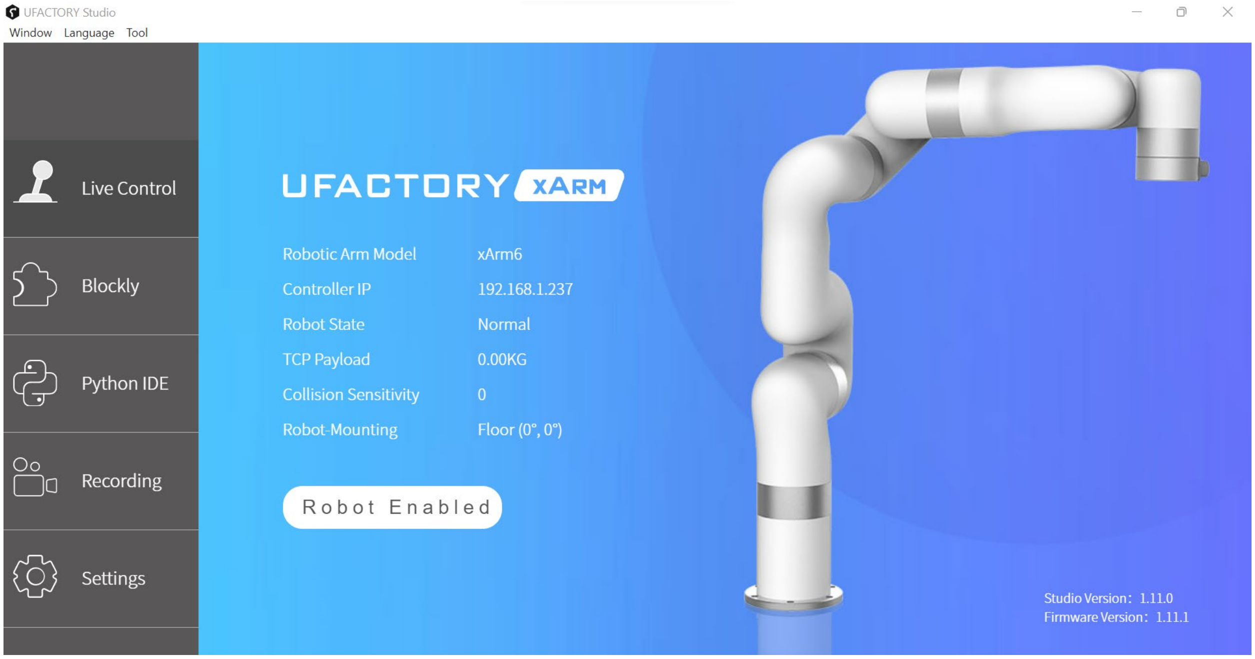

Figure 45. Image of the main menu of the UFACTORY Studio software.

The IP address of the control box must also be added to the STAR System python code. On line 38 (Figure 46), replace “XXX.XXX.X.XXX” with the IP address of the control box.

```
37    # Set up the xarm
38    arm = XArmAPI('XXX.XXX.X.XXX', baud_checkset=False)# Set the arm address, NOTE: NEED TO EDIT
```

Figure 46. Image of the line in the STAR System python code that must be edited with the IP address of the control box.

One-time Calibrations

There are a couple of steps that will need to be taken upon first powering up the robot and linear motor. The first of which is initializing the linear motor. Navigate from the main menu of the UFACTORY Studio software (Figure 45) to the Settings menu. From there, go to Tools > Linear Motor (Figure 47). Select the Initialize button. This initialization process will move the robot

along the track until the sensors on the robot mounting platform and the end of the linear motor detect each other, which allows the linear motor to determine where both end points of the track are located. During this process, ensure the movement path of the robot remains clear of obstructions.

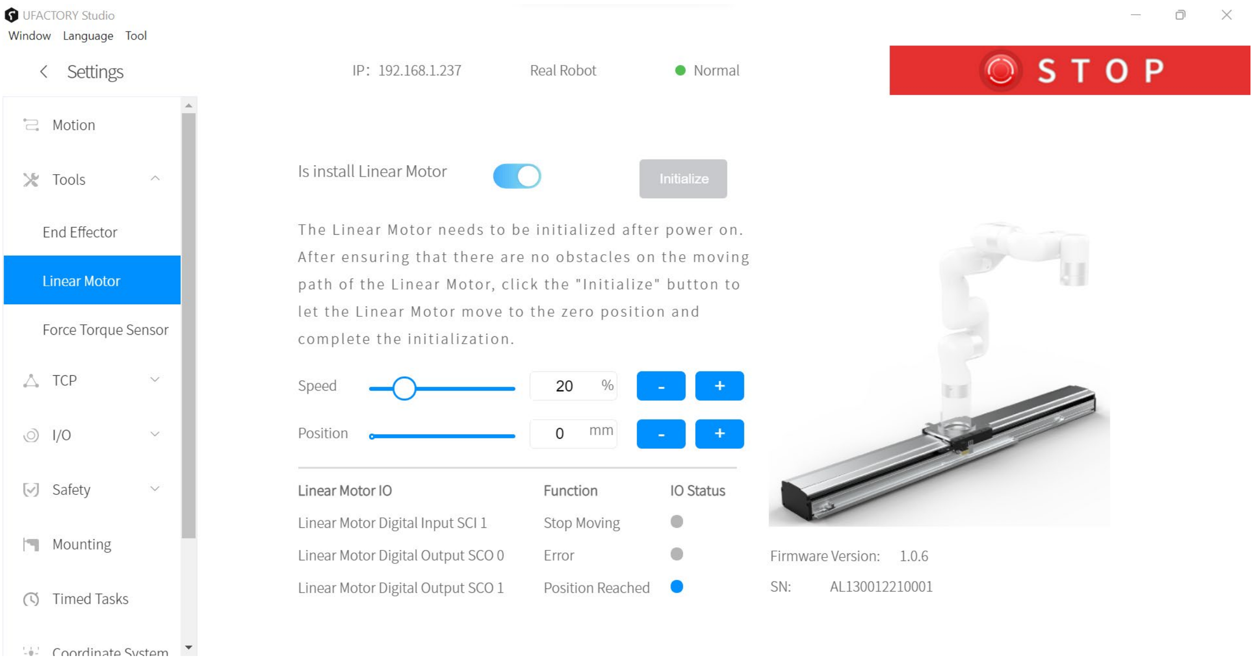

Figure 47. Image of the Linear Motor settings screen.

The robot will also need to undergo a friction test the first time it is used. During this calibration process, the robot will move at various angles and speeds to determine the latent friction experienced by the robot while it is unburdened, which has implications for the robot's ability to detect collisions. For this test, the robot will need a one meter cube of area around it that is completely free of obstructions. Any obstructions in that area may be struck by the robot if this routine is initiated. In the settings menu, navigate to Advanced > Advanced Tools > Friction Identification (Figure 48). Read the warning that pops up and ensure the system is compliant with the requirements it provides, and select start. Once the routine is done and the progress bar reaches 100%, close out of the dialogue box.

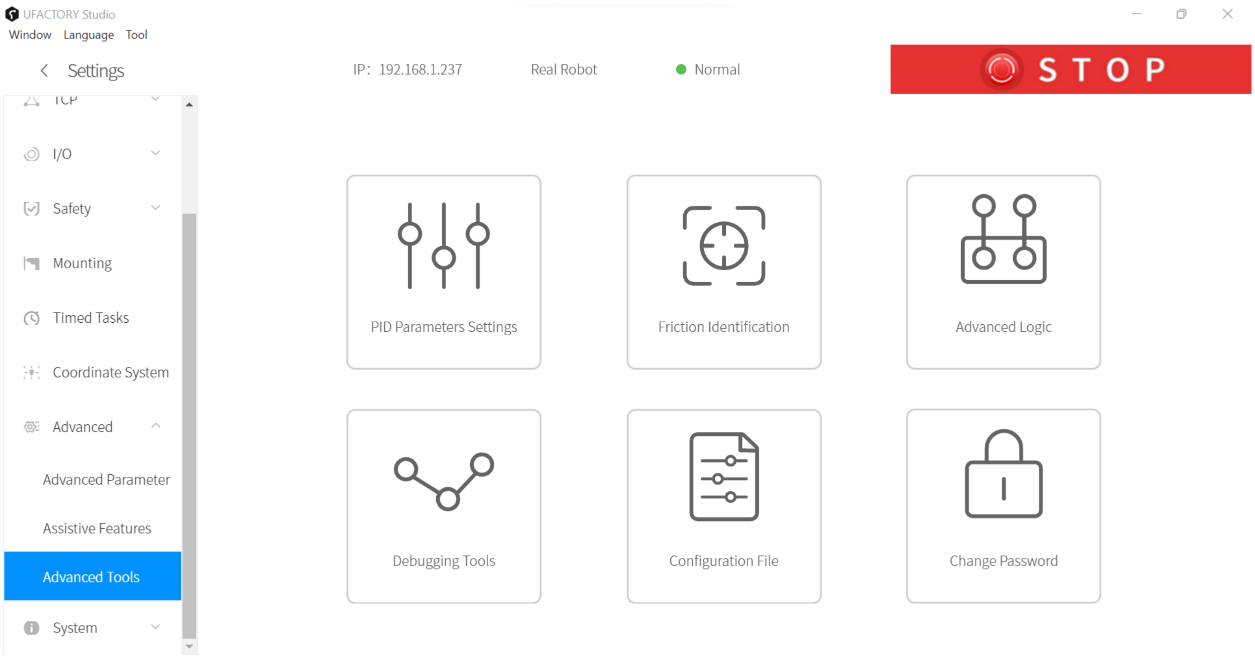

Figure 48. Image highlighting the Friction Identification routine.

Upon completion of the Friction Identification routine, navigate to the Motion section (Figure 49). Under Sensitivity Setting, there is a slider for Collision Sensitivity. This determines how much force is required in order to set off the torque sensor error, where less sensitivity requires more force to activate the error. The tubing that is strung along the robot may cause enough friction to activate the error if the sensitivity is too high, so adjustment may be required to this setting for the system to work properly.

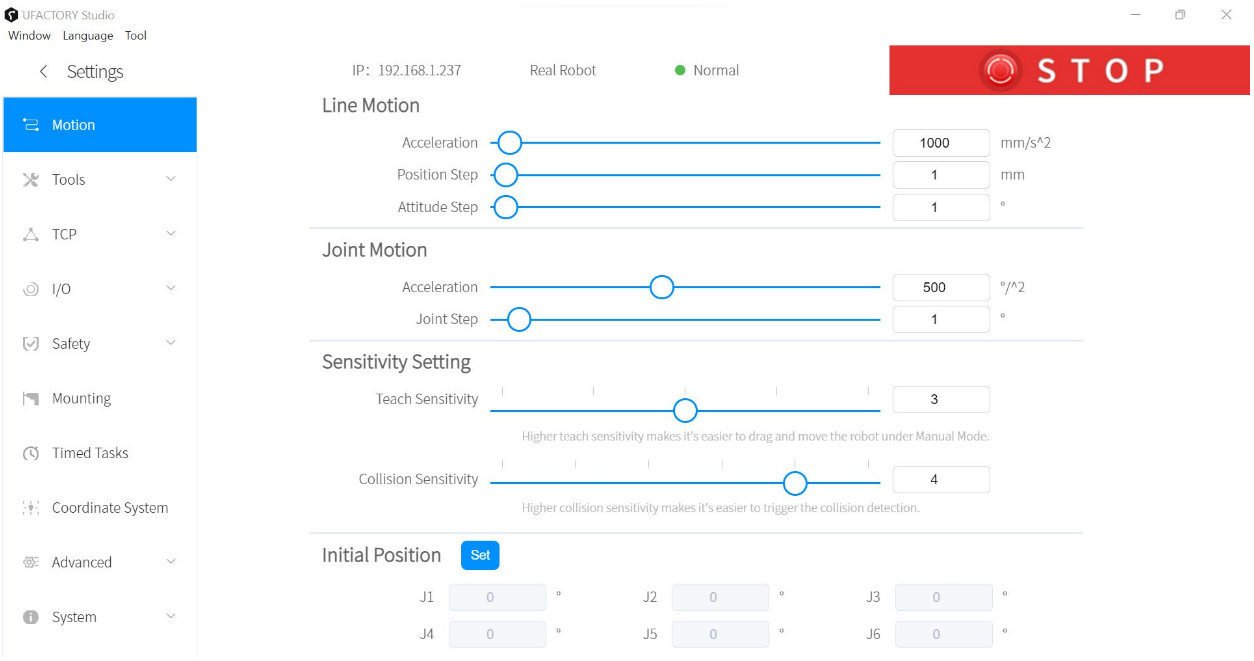

Figure 49. Image highlighting the collision sensitivity setting.

Position Calibration

The positioning of the robotic arm above the sample vessels is determined by a series of X, Y, and Z coordinates as well as roll, pitch, and yaw angles in the STAR System python code. These coordinates in the python code are specific to the original STAR System and must be adjusted to the specifics of the new system being built.

Starting on line 48 in the python code (Figure 50) is an array of 48 individual positions. Each line corresponds to the position indicated in the comment next to the array. The robot moves through these positions sequentially starting with position 1 and ending with position 48. The first three numbers of each position in the array relate to X, Y, and Z coordinates centered on the base of the robot. Each of these coordinates should be edited to correspond to the desired dosing location of the position.

```python
# Define position array
posArray =[[0, 0, 0, 0, 0, 0],
            [350.0, -100.0, 110.0, 180.0, 0.0, 0.0],# pos1
            [450.0, -100.0, 110.0, 180.0, 0.0, 0.0],# pos2
            [550.0, -100.0, 110.0, 180.0, 0.0, 0.0],# pos3
            [650.0, -100.0, 110.0, 180.0, 0.0, 0.0],# pos4
            [650.0, 100.0, 110.0, 180.0, 0.0, 0.0],# pos5
            [550.0, 100.0, 110.0, 180.0, 0.0, 0.0],# pos6
            [450.0, 100.0, 110.0, 180.0, 0.0, 0.0],# pos7
            [350.0, 100.0, 110.0, 180.0, 0.0, 0.0],# pos8
            [350.0, 200.0, 110.0, 180.0, 0.0, 0.0],# pos9
            [450.0, 200.0, 110.0, 180.0, 0.0, 0.0],# pos10
            [550.0, 200.0, 110.0, 180.0, 0.0, 0.0],# pos11
            [650.0, 200.0, 110.0, 180.0, 0.0, 0.0],# pos12
            [650.0, 300.0, 110.0, 180.0, 0.0, 0.0],# pos13
            [550.0, 300.0, 110.0, 180.0, 0.0, 0.0],# pos14
            [450.0, 300.0, 110.0, 180.0, 0.0, 0.0],# pos15
            [350.0, 300.0, 110.0, 180.0, 0.0, 0.0],# pos16
```

Figure 50. Image of the position array in the STAR System python code.

To find what coordinates to input in the position array, open the “Live Control” tab of the main UFACTORY Studio menu (Figure 51). Select the “MANUAL MODE” button so it highlights blue and says “ON”. This allows for the physical manipulation of the robot into the desired angles and locations. Once the robot is correctly oriented for a given position, observe the “Position” and “Orientation” values in the bottom left corner of the screen. Replace the default values in the position array with these new values. Repeat this process for every position.

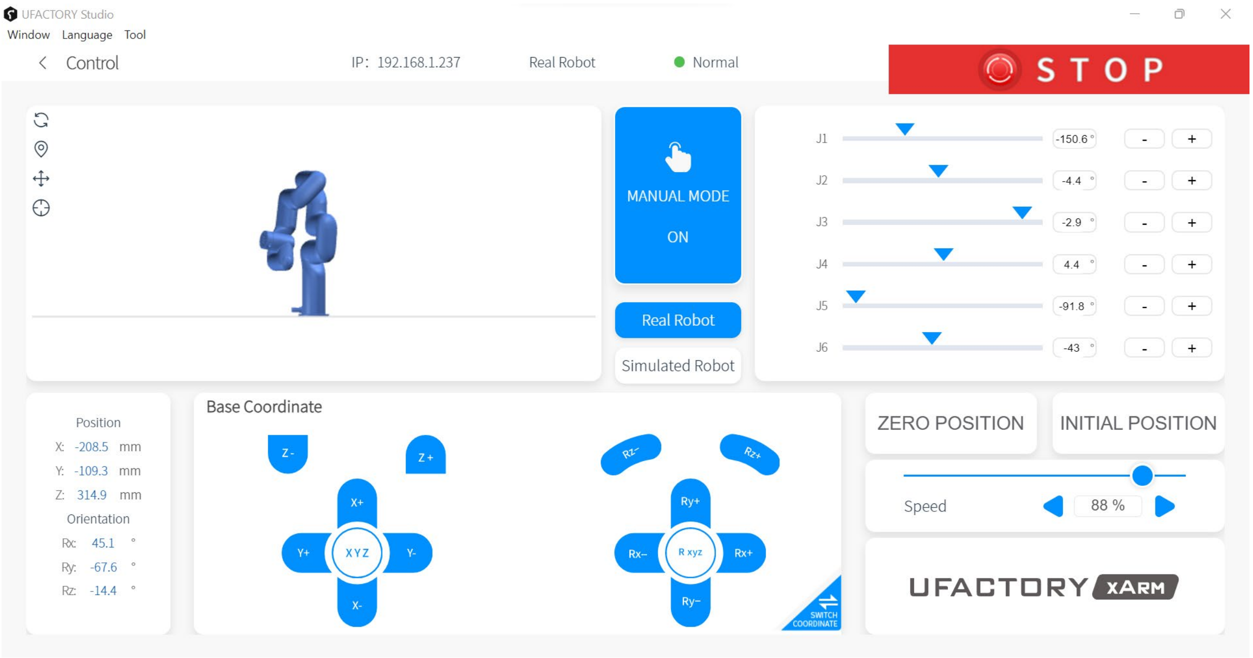

Figure 51. Image of the Live Control section of the UFACTORY Studio software.
