# Supplementary material for: Sequential Treatment Application Robot (STAR) for high-replication marine experimentation
Source: HardwareX. 2024 Mar 28;18:e00524. doi: 10.1016/j.ohx.2024.e00524 (PMC11022082; doi:10.1016/j.ohx.2024.e00524)
Supplement: Supplementary data 2 [file mmc2.docx]

| **Source file repository** | **DOI: 10.5281/zenodo.10048123**  **https://zenodo.org/doi/10.5281/zenodo.10048123** |
| --- | --- |

***Design files***

1. **Design files summary**

| **Design file name** | **File type** | **License** | **Location** |
| --- | --- | --- | --- |
| starProgram.zip | Labview code | CC BY 4.0 | Source file repository |
| starArmControl.py | Python code | CC BY 4.0 | Source file repository |
| starWatchdogCode.txt | Particle.io code | CC BY 4.0 | Source file repository |
| starThingspeakCode.txt | Matlab code | CC BY 4.0 | Source file repository |
| starStirCode.ino | Arduino code | CC BY 4.0 | Source file repository |
| star3dPrintFiles.zip | STL files | CC BY 4.0 | Source file repository |
| starLaserCutFiles.zip | Vector files | CC BY 4.0 | Source file repository |
| stirControlBoard.zip | Eagle files | CC BY 4.0 | Source file repository |
| starBillOfMaterials.xlsx | MS Excel file | CC BY 4.0 | Source file repository |

**starProgram.zip** is the Labview code that runs the user interface, the peristaltic pumps, and the syringe pump. It calls the Python functions that control the arm position and relays information to the cellular watchdog. The starSystem.lvproj is the Labview project file that contains the main program (virtual instrument, VI) named starSystemControl.vi and maintains all of the proper references among subvi’s.

**starArmControl.py** is the Python code for controlling the robotic arm movement.

**starWatchdogCode.txt** is the Particle.io code that is uploaded to the Boron cellular-enabled microcontroller.

**starThingspeakCode.txt** is the Matlab script that summarizes data sent from Particle.io to ThingSpeak.

**starStirCode.ino** is the Arduino code that runs the stir plate controller.

**star3dPrintFiles.zip** is a zipped folder that contains all of the CAD files for 3D printing the custom components of the STAR. It is organized into folders as follows: doserSystem (fluidRouting, pumpHousing), endEffector, stirSystem (sampleContainment, stirController, stirPlate), watchdog.

**starLaserCutFiles.zip** is a zipped folder that contains the vector files for laser cutting the custom acrylic components of the STAR. It includes two vector files with the parts arranged on them, each is intended to be cut on a 24x24in piece of ¼in acrylic.

**starStirControlBoard.zip** is a zipped folder that contains the Eagle (Autodesk) board and schematic files used to create the stir controller circuit board.

1. **Bill of materials summary**

The detailed bill of materials is available as an Excel spreadsheet (starBillOfMaterials.xls) in the source file repository. It is organized by system and subsystem, where applicable. Costs are approximate and sources represent an option that was available in our purchasing systems at the time of development, rather than a suggestion on the part of the authors. It is likely that similar parts can be sourced for cheaper. Custom 3D printed parts are priced according to estimated material costs, as prices for fabrication can be highly variable.

1. **Build instructions**

Build instructions are provided as a pdf document (starBuildManual.pdf) in the source file repository. Additional details on connecting the STAR subsystems can be found in the hardware description and associated figures.
